# Downregulation of NAD Kinase Expression in β‐Cells Contributes to the Aging‐Associated Decline in Glucose‐Stimulated Insulin Secretion

**DOI:** 10.1111/acel.70037

**Published:** 2025-03-05

**Authors:** Guan‐Jie Li, Mei‐Ling Cheng, Yu‐Ting Lin, Yu‐Hsuan Ho, Gigin Lin, Chih‐Yung Chiu, Hung‐Yao Ho

**Affiliations:** ^1^ Graduate Institute of Biomedical Sciences, College of Medicine Chang Gung University Taoyuan Taiwan; ^2^ Metabolomics Core Laboratory, Healthy Aging Research Center Chang Gung University Taoyuan Taiwan; ^3^ Clinical Metabolomics Core Laboratory Chang Gung Memorial Hospital at Linkou Taoyuan Taiwan; ^4^ Department of Biomedical Sciences, College of Medicine Chang Gung University Taoyuan Taiwan; ^5^ School of Physical Therapy, College of Medicine Chang Gung University Taoyuan Taiwan; ^6^ Department of Medical Imaging and Intervention Chang Gung Memorial Hospital at Linkou and Chang Gung University Taoyuan Taiwan; ^7^ Department of Medical Imaging and Radiological Sciences Chang Gung University Taoyuan Taiwan; ^8^ Department of Pediatrics Chang Gung Memorial Hospital at Linkou, and Chang Gung University Taoyuan Taiwan; ^9^ Department of Pediatrics Chang Gung Memorial Hospital at Keelung, and Chang Gung University Taoyuan Taiwan; ^10^ Department of Medical Biotechnology and Laboratory Science, College of Medicine Chang Gung University Taoyuan Taiwan; ^11^ Research Center for Emerging Viral Infections Chang Gung University Taoyuan Taiwan

**Keywords:** glucose‐stimulated insulin secretion (GSIS), NADK, NADK2, pancreatic β‐cells

## Abstract

Nicotinamide adenine dinucleotide kinase (NADK) is essential to the generation of nicotinamide adenine dinucleotide phosphate (NADP(H)), an important metabolic coupling factor involved in glucose‐stimulated insulin secretion. In the present study, we showed that the expression of *Nadk* and *Nadk2* transcripts and NADP(H) content were lower in islets of 80‐week‐old (*aged*) mice than those of 8‐week‐old (*young*) mice. This was associated with diminished oral glucose tolerance of old mice and the glucose‐stimulated insulin secretion (GSIS) response of islets. Knockdown (KD) of *Nadk* or *Nadk2* gene expression in NIT‐1 cells impaired glucose‐stimulated insulin secretion. Metabolomic analysis revealed that *Nadk* KD specifically affected purine metabolism in glucose‐stimulated cells. The levels of 5‐aminoimidazole‐4‐carboxamide ribonucleotide (AICAR) were higher in KD cells than in the non‐targeting control (NTC) cells. Phosphorylation of AMP‐activated protein kinase (AMPK) was elevated in glucose‐treated KD cells compared to that of NTC cells. Increased AICAR level and AMPKα phosphorylation were observed in the glucose‐stimulated islets of the aged mice. Genetic and pharmacological inhibition of AMPK promoted glucose‐stimulated insulin release by KD cells and the aged mouse islets. It is likely that NADK is modulatory to AMPK activation in pancreatic β‐cells and to their GSIS response. Enhanced AICAR formation in KD cells was accompanied by significantly increased conversion from inosine monophosphate (IMP) in a tetrahydrofolate (THF)‐dependent manner. Folate supplementation augmented the GSIS response of KD cells and aged mouse islets. Taken together, these findings suggest that the aging‐associated decline in NADK expression may underlie the reduced insulin secretory capacity of pancreatic β‐cells.

## Graphical abstract

Adequate NADK/NADK2 level is essential to the proper functioning of redox and folate cycles and the purine biosynthesis pathway, and as well as maintenance of GSIS response. During aging, diminished NADK/NADK2 expression alters the metabolic status quo, increasing AICAR production. The latter activates AMPK and suppresses GSIS.

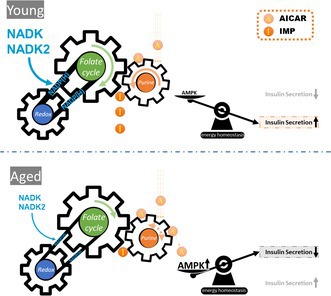

**Abbreviations**
AICAR: 5‐aminoimidazole‐4‐carboxamide ribonucleotide
ATIC: 5‐aminoimidazole‐4‐carboxamide ribonucleotide formyltransferase/inosine monophosphate cyclohydrolase
FAICAR: 5‐formamidoimidazole‐4‐carboxamide ribotide
GEO: Gene Expression Omnibus
GSIS: glucose stimulated insulin secretion
NAD: nicotinamide adenine dinucleotide
NADK: nicotinamide adenine dinucleotide kinase
NADP: nicotinamide adenine dinucleotide phosphate
OGTT: oral glucose tolerance test
shRNA: short hairpin RNA
siRNA: small interfering RNA

## 1 Introduction

Secretion of insulin by β‐cells depends on the coupling of metabolic activity to the secretory process in a process known as metabolic coupling (Prentki et al. 2013). A number of metabolic intermediates and coenzymes, termed metabolic coupling factors (MCFs), are involved in the regulation of the process and the triggering of insulin granule exocytosis. These MCFs include adenine and guanine nucleotides, citrate, malonyl‐coenzyme A (CoA), cyclic adenosine monophosphate, glutamate, monoacylglycerol, short‐chain acyl‐CoA, and etc. (Prentki et al. 2013). Nicotinamide adenine dinucleotide (NAD) and its phosphate form NADPH are important MCFs. It was postulated that NADH increases ATP and reactive oxygen species (ROS) generation via the donation of its electrons to the electron transport chain (ETC). Both ATP and ROS are needed for insulin secretion (Luo et al. 2015). An alternative nonexclusive view is that NADH can be converted to NADPH through pyruvate–malate, pyruvate–citrate, and pyruvate–citrate pathways (Luo et al. 2015). NADPH is harnessed for glutaredoxin‐1 (GRX‐1)‐mediated reduction of disulfide bonds of target proteins involved in insulin secretion (Huypens et al. 2012). Change in the NADPH/NADP^+^ ratio is modulatory to the properties of voltage‐dependent K^+^ channel Kv2.1, which is implicated in the repolarization of β‐cell membrane (MacDonald et al. 2003). Recently, a model of metabolic and redox oscillations entailed in β‐cell glucose sensing, known as the Mito_Cat_–Mito_Ox_ model, has been proposed (Merrins et al. 2022). Cyclic changes in ROS and NADPH regulate insulin exocytosis. NADPH is proposed to serve as “off switch” signal to reset metabolic oscillations and insulin secretion. However, NADPH generated by the reductive tricarboxylic acid (TCA) cycle activates sentrin/SUMO‐specific protease‐1 (SENP1), which causes de‐SUMOylation of molecular targets to promote insulin exocytosis (Zhang, Jensen, et al. 2021). It appears that the roles of NADPH in metabolic signaling are more complicated than previously thought.

NADP(H) is generated through the de novo biosynthesis or salvage pathways. Quinolinic acid is formed from tryptophan in mammals and is converted to nicotinic acid mononucleotide (NAMN). The latter is acted upon by nicotinamide/nicotinic acid mononucleotide adenylyltransferase (N(A)MNAT) to form nicotinic acid adenine dinucleotide (NAAD) and is further amidated to NAD through the action of NAD synthetase (Pollak et al. 2007). Nicotinic acid and nicotinamide, which are themselves the degradation products of NAD(P), can be salvaged to NAMN and nicotinamide mononucleotide (NMN) by phosphoribosyltransferase activities for NAD synthesis. NAD is phosphorylated by NAD kinase (NADK) to generate NADP. There are two human NADK isoforms, namely the cytosolic NADK and mitochondrial NADK2 (Zhang and Zhang 2023). Knockdown of *Nadk* gene expression diminished the NADP(H) pool in β‐cells and hampered their insulin secretion (Gray et al. 2012), suggesting regulatory roles for NADK and cytosolic NADP(H) in β‐cell metabolic signaling. It is unclear whether NADK2 plays similar roles in β‐cell physiology.

Age is known to be a risk factor for diabetes mellitus (de Tata 2014; Ketut et al. 2012). The functions and proliferative potential of β‐cells decline with aging (Aguayo‐Mazzucato 2020; Tuduri et al. 2022). The levels of NAD(H) and NADP(H) were lower in most tissues of the aged mice than those of young mice (McReynolds et al. 2021). Additionally, the fluorescence lifetime imaging (FLIM) showed that the level of protein‐bound NAD(P)H decreased in the islets of aged mice versus those of young mice (Gregg et al. 2016). It is plausible that the aging‐related decreases in nicotinamide adenine dinucleotides may be associated with impairment of β‐cell function and insulin secretion.

Purines are formed via de novo biosynthetic or salvage pathways. The de novo purine biosynthetic pathway consists of ten reactions converting phosphoribosyl pyrophosphate to inosine 5′‐monophosphate (IMP). The enzymes involved are thought to form clusters known as purinosomes that are localized near mitochondria (Pedley et al. 2022). It has been recently postulated that the purine metabolic pathway is associated with the onset of diabetes, and certain elements may serve as early diagnostic biomarkers (Hameed et al. 2023). Framingham Heart Study Generation 3 revealed that uric acid, a degradation product of hypoxanthine, showed significant correlation with the body mass indices (BMI), the fasting glucose levels, and the homeostasis model assessment‐insulin resistance index (HOMA‐IR) values of the study participants (Kimberly et al. 2017). The activity of xanthine oxidoreductase correlated positively with BMI, fasting plasma insulin, and HOMA‐IR among the type 2 diabetes patients (Okuyama et al. 2021). Additionally, adenylosuccinate can stimulate insulin release by β‐cells (Gooding et al. 2015). These findings imply that purine metabolites play important roles in glucose homeostasis, and anomalous purine metabolism may contribute to the pathogenesis of diabetes.

In the current study, we demonstrated that the pancreatic islets of aged mice express fewer *Nadk* and *Nadk2* transcripts and have a lower GSIS response than those of their young counterparts. NADK deficiency causes differential changes in purine metabolism during high‐glucose stimulation of β‐cells. AICAR accumulation may lead to AMPK activation and inhibition of insulin release. Similar biochemical and physiological changes occur in the pancreatic islets of aged mice with reduced *Nadk* and *Nadk2* gene expression. Diminution of NADP(H) pool and THF cycling in *Nadk* KD or *Nadk2* KD cells promotes the formation of AICAR from IMP. Folate supplementation boosts THF cycling and GSIS response.

## 2 Materials and Methods

### 2.1 Animal Study

The male C57BL/6JNarl mice were available from the National Laboratory Animal Center and reared in individually ventilated cages in the specific pathogen‐free (SPF) facility of the Chang Gung University Laboratory Animal Center. Mice were fed a chow diet (LabDiet 5010) and maintained under a light/dark cycle (12/12 h). The body weight of the mice was recorded weekly (Figure S1).

For the oral glucose tolerance test (OGTT), the mice were fasted for 6 h and administered a bolus of glucose (2 g/kg of body weight) via oral gavage. Blood was collected from the tail vein at 0, 15, 30, 60, and 120 min after glucose administration. The blood glucose concentration was determined with a glucometer. Two microliters of blood was applied to Accu‐Chek Guide glucose test strips (Roche Diabetes Care; Indianapolis, IN, USA), and the glucose concentration was measured with the Accu‐Chek Guide Me glucometer (Roche Diabetes Care). Additionally, 10 μL plasma was analyzed for insulin levels.

### 2.2 Cell Culture

NIT‐1 mouse insulinoma cells (American Type Culture Collection (ATCC) number: CRL‐2055; Research Resource Identifier (RRID): CVCL_3561) were cultured in Ham's F12K medium/2 mM glutamine/10% fetal bovine serum at 37°C in a humidified atmosphere containing 5% CO_2_.

For GSIS assay of β‐cells, 2 × 10^6^ cells were seeded in a 6‐well cell culture plate. Forty‐eight hours later, cells were washed twice with Krebs‐Ringer bicarbonate buffer (KRBB; 10 mM HEPES/118.5 mM NaCl/2.54 mM CaCl_2_/1.19 mM KH_2_PO_4_/4.74 mM KCl/25 mM NaHCO_3_/1.19 mM MgSO_4_) and incubated in KRBB containing 0.1% bovine serum albumin (BSA) for 5 h. Cells were washed twice with KRBB and then treated with KRBB/0.1% BSA supplemented with 2 mM glucose (*low glucose*) or 16.5 mM glucose (*high glucose*) for 1 h. The medium was collected for the determination of insulin using the Mercodia Mouse Insulin ELISA kit (catalog number: 10‐1247‐01; Mercodia, Sweden) according to the manufacturer's instructions.

### 2.3 Islet Isolation

The murine pancreatic islets were isolated as previously described with modifications (Bertera et al. 2012; Carter et al. 2009; Corbin et al. 2021; Li et al. 2009). Briefly stated, mice were fasted for 12 h before islet isolation and anesthetized with isoflurane (1 μL/g body weight). The mouse was then sacrificed, and the abdominal skin was cut open to expose the viscera. About 2–3 mL of cold collagenase (1.5 mg/mL in Hanks' balanced salt solution, HBSS) was injected into the common bile duct with a 30G needle till the pancreas was distended. The pancreas was removed and placed in a 50 mL conical tube containing 2 mL of cold collagenase solution. The tube was incubated at 37°C for 20 min. About 30 mL Roswell Park Memorial Institute (RPMI) 1640 medium supplemented with 10% fetal bovine serum (FBS) was added to stop the collagenase digestion. The sample was centrifuged at 300×*g* for 5 min at 4°C. The pellet was resuspended in HBSS, filtered, and centrifuged. The pellet was resuspended in Histopaque‐1077 (SI‐10771; Sigma‐Aldrich, St. Louis, MO, USA), and an equal volume of RPMI 1640 medium/10% FBS was added. The suspension was centrifuged at 1200×*g* for 25 min at 4°C. The islets were retrieved from the interface between two layers and resuspended in 15 mL of RPMI 1640 medium/10% FBS. The suspension was centrifuged and washed three times with HBSS. After washing, the pellet was resuspended in 10 mL RPMI 1640 medium/10% FBS and transferred to a tissue culture dish. The islets were handpicked and maintained in RPMI 1640 medium/10% FBS at 37°C in a humidified atmosphere containing 5% CO_2_.

For GSIS assay of pancreatic islets, the islets were cultured in RPMI 1640 medium/10% FBS for 1 day (d), washed twice with KRBB, and incubated in KRBB/0.1% BSA for 1 h. They were further washed with KRBB twice and treated with KRBB/0.1% BSA supplemented with 2 mM glucose (*low glucose*) or 16.5 mM glucose (*high glucose*) for 1 h. The medium was collected for determination of insulin using ELISA.

### 2.4 Molecular Biology Techniques

For RNA isolation, the cells or the islet pellet were lysed in TRIzol (Thermo Fisher Scientific, Waltham, MA, USA) according to the manufacturer's instruction. The cDNA was synthesized, and reverse transcription‐quantitative polymerase chain reaction (RT‐qPCR) was performed as previously described (Cheng et al. 2022). The primers used are tabulated in Table S1.

The lentiviral vectors encoding the shRNAs against *Nadk* (TRCN0000297518), *Nadk2* (TRCN0000024982), *Prkaa1* (TRCN0000360841) and *Prkaa2* (TRCN0000360846), and the non‐targeting control (NTC, NTC2) lentiviral vectors were acquired from the RNA Technology Platform and Gene Manipulation Core Laboratory (Academia Sinica, Taipei, Taiwan). The lentiviruses were packaged as described previously (Cheng et al. 2022). The virus‐containing supernatant was added to NIT‐1 cells in the presence of 8 μg/mL polybrene. The transduced cells were selected in the medium containing 2 μg/mL puromycin.

For transfection of islets with siRNA, the islets were treated for transfection as previously described with modifications (Rabinovitch et al. 1999). Briefly stated, the islets were transfected with 25 pmole of siRNA duplex using Lipofectamine RNAiMAX reagent (Thermo Fisher Scientific) according to the manufacturer's instructions. One day later, the islets were harvested for transcript quantification and GSIS assay. The siRNA duplex for knockdown of *Prkaa1* gene expression was a mixture of s98535 and s98536 (Mission Predesigned siRNA pooled product, Sigma‐Aldrich), while that for knockdown of *Prkaa2* gene expression was a mixture of s99117 and s99116. The predesigned universal non‐targeting control siRNA duplex (NTC3) was also available from Sigma‐Aldrich.

### 2.5 Metabolomic Analysis

Metabolites were extracted as previously described with modifications (Cheng et al. 2021). Cells were scraped in 1 mL 55% methanol (prechilled at −80°C). The resulting extract was vortexed and centrifuged at 12,000×*g* for 30 min at 4°C. The supernatant was mixed with 4 volumes of acetonitrile (ACN), vortexed for 30 s, and centrifuged. The supernatant was retained and dried under nitrogen gas. The islets were collected by centrifugation at 300×*g* for 5 min at 4°C. The pellet was washed twice by resuspension in phosphate‐buffered saline (PBS) and centrifugation. After the final wash, the sample was resuspended in 1 mL 55% methanol, vortexed for 3 min, and centrifuged at 12,000×*g* for 30 min at 4°C. The supernatant was mixed with ACN and processed as described for extraction of cellular metabolites.

For liquid chromatography‐mass spectrometric (UPLC–MS) analysis of cellular metabolites, an ACQUITY UPLC HSS T3 column (2.1 mm × 100 mm; particle size: 1.8 μm) (Water Corp., Milford, MA, USA) was used. Chromatographic separation was maintained at a flow rate of 400 μL/min and a temperature of 40°C. The following elution profile was used: 0–4 min, 1%–50% B; 4–5 min, 50%–98% B; 5–7.4 min, 98% B; 7.4–7.5 min, 98%–1% B; and 7.5–10 min, 1% B. Mobile phase A was 0.1% formic acid (FA), and mobile phase B was 0.1% FA/ACN. Mass spectrometric (MS) analysis was performed using a Waters SYNAPT G2‐S HDMS TOF‐MS (Waters Corp.). The desolvation gas flow was set at 1000 L/h at a temperature of 500°C, and the source temperature was 150°C. The capillary voltage and cone voltage were set at 3 kV and 25 V, respectively.

The untargeted metabolomics data were analyzed as previously described (Cheng et al. 2020). For validation of metabolites, the standard compounds were analyzed in parallel. For untargeted metabolomics data, the false discovery rate (FDR) with Bonferroni correction was applied. Metabolites with an FDR adjusted *q*‐value less than 0.05 were considered to be differentially abundant. For targeted analysis of the metabolites in islets, chromatographic separation on an ACQUITY BEH amide column (2.1 mm × 100 mm; particle size: 1.7 μm) (Water Corp., Milford, MA, USA) was performed at a flow rate of 300 μL/min and a temperature of 45°C. The MS analysis was performed similarly, with exceptions to certain parameters. The capillary voltage and cone voltage were adjusted to 2 kV and 5 V, respectively. The quantitative measurement of AICAR was performed in a similar way with the use of a reference standard compound.

For quantitative measurement of folate derivatives, the cells were washed twice with PBS and scraped in 50% methanol/10 g/L NH_4_HCO_2_/5 g/L ascorbic acid (pH 3.2). The sample was vortexed, centrifuged at 12,000×*g* for 15 min at 4°C, and purified using a solid phase extraction (SPE) cartridge (Bond Elut‐PH #12102032; Agilent Technologies Inc., Santa Clara, CA, USA). The sample was eluted with 40% methanol/10% ACN/0.5% CH_3_COOH and dried under nitrogen gas. The LC–MS analysis was conducted as described for the targeted metabolite analysis of islets. The reference standard compounds were analyzed in parallel for quantitative purposes.

### 2.6 [ ^15^N_4_ ]‐Inosine Labeling and Analysis

For analysis of [^15^N_4_]‐inosine‐labeled cells, cells were incubated in Ham's F12K medium/2 mM glutamine/10% fetal bovine serum, which was supplemented without or with 80 μM [^15^N_4_]‐inosine for 48 h. The labeled cells were treated with glucose under the condition that mimicked GSIS, as described earlier. They were harvested at 0, 12, and 20 min post‐stimulation and extracted with 55% methanol. The sample was chromatographed on an ACQUITY UPLC HSS CSH C18 (2.1 × 100 mm; particle size: 1.7 μm), which was maintained at 45°C. The flow rate was set at 300 μL/min. The following elution profile was adopted: 0–6 min, 2% B; 6–6.1 min, 2%–25% B; 6.1–9 min, 25%–30% B; 9–9.8 min, 30%–50% B; 9.8–12 min, 50% B; 12–12.1 min, 50%–2% B; and 12.1–15 min, 2% B. Mobile phase A was 0.1% FA, and mobile phase B was ACN. The MS analysis was carried out as described in the preceding section with the following exceptions: the desolvation gas flow was set at 800 L/h; the capillary voltage was set at 1.5 kV.

### 2.7 Analysis of NADP(H)

Cells were extracted in 1 mL 55% methanol/10 mM KOH and centrifuged at 12,000×*g* for 15 min at 4°C. The supernatant was mixed with 4 volumes of ACN and centrifuged again. The supernatant was retained and chromatographed on an ACQUITY UPLC HSS T3 column (2.1 mm × 150 mm; particle size: 1.8 μm) (Water Corp.) that was maintained at 37°C. The flow rate was set at 380 μL/min. An elution profile was used: 0–2 min, 0% B; 2–2.5 min, 0%–3% B; 2.5–5 min, 3%–4% B; 5–7.5 min, 4%–15% B; 7.5–8.5 min, 15% B; 8.5–9.5 min, 15%–0% B; and 9.5–11 min, 0% B. Mobile phase A was 25 mM KH_2_PO_4_ (pH 5.8), and mobile phase B was methanol. The reference standard compounds were analyzed in parallel for the quantitative purpose.

### 2.8 Immunological Techniques

The mice at the age of 8 and 80 weeks were fasted and fed an oral bolus of glucose (2 g/kg of body weight). At the indicated times, the pancreas was dissected out and snap frozen in OCT medium over liquid nitrogen. The frozen tissue was cryosectioned to a thickness of 7 μm. The section was processed for immunohistochemical (IHC) detection with anti‐phospho‐AMPKα (40H9; #2535; Cell Signaling Technology (CST), MA, USA), anti‐total AMPKα (bs‐1115R; Bioss Antibodies, MA, USA), anti‐insulin (tcba7238; Taiclone, Keelung, Taiwan), and horseradish peroxidase (HRP)‐conjugated goat anti‐rabbit IgG (C04003; Croyez, Taipei, Taiwan) antibodies according to CST instructions with modifications. The primary antibodies were used at a 1:200 dilution in PBS/0.1% Tween 20 (PBST) containing 2.5% bovine serum albumin (BSA), and the secondary antibody was used at a 1:100 dilution. 3,3′‐Diaminobenzidine (DAB) solution (0.05% DAB/0.015% H_2_O_2_/PBS) was used as a substrate for color development. The section was counterstained with hematoxylin.

The western blotting was performed as previously described (Cheng et al. 2023). The primary antibodies, including anti‐phospho‐AMPKα (40H9; #2535; CST) and anti‐AMPKα (23A3; #2603; CST) antibodies, were used at the dilution of 1:1000 in PBST/5% BSA, and HRP‐conjugated goat anti‐rabbit IgG (C04003; Croyez) antibody was used at the dilution of 1:10,000.

### 2.9 Statistical Analyses

All statistical analyses were conducted using GraphPad Prism 7 software (GraphPad Software Inc., CA, USA). Two‐way analysis of variance (ANOVA) with Sidak's multiple comparison test, Mann–Whitney test, Kruskal–Wallis test with Dunn's multiple comparison test, and Student's *t* test were used where appropriate.

## 3 Results

### 3.1 Association of the Age‐Related Decline in Pancreatic Islet Function and NADP(H) With *Nadk* and *Nadk2* Gene Expression

**FIGURE 1:**
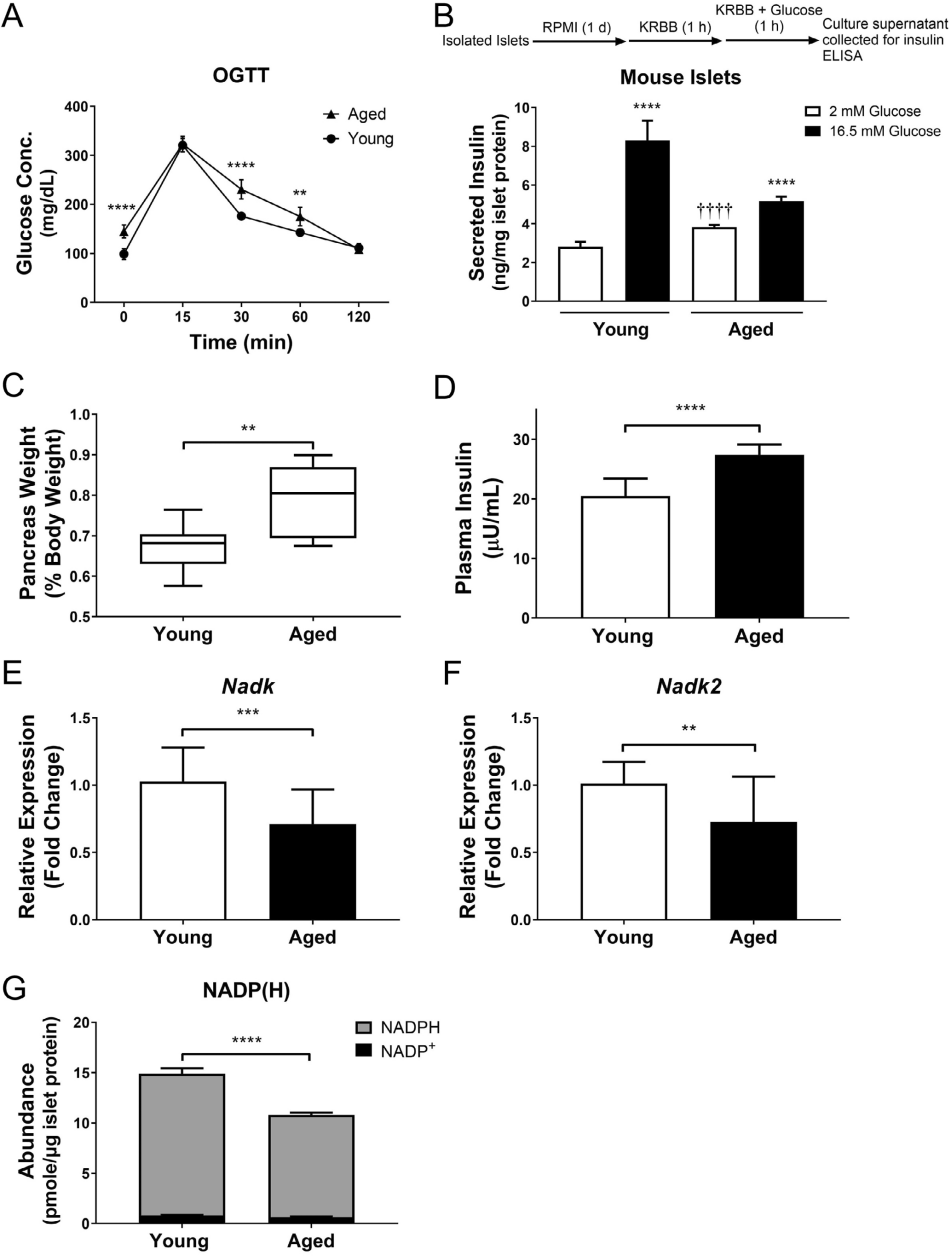
Decline in glucose homeostasis during aging is associated with reduced *Nadk* and *Nadk2* expression. (A) Blood glucose concentrations of the 80‐ (*aged*) and 8‐ (*young*) week‐old mice were determined at indicated time points after glucose administration in OGTT. Data are mean ± SD (*N* = 6). **p < 0.01, ****p < 0.001, versus aged mice. (B) Islets isolated from the aged and young mice were treated with 2 or 16.5 mM glucose, and the culture supernatants were collected for insulin determination. Data are mean ± SD (*N* = 6). ****p < 0.001, 2 versus 16.5 mM glucose treatment; ^††††^ *p* < 0.001, versus similarly treated young mouse islets. (C) Relative weights of pancreata of the aged and young mice (% body weight) are shown. Data are mean ± SD (*N* = 6). **p < 0.01, aged mice versus young mice. (D) Fasting plasma insulin levels (μU/mL) of the aged and young mice are shown. Data are mean ± SD (*N* = 9). ****p < 0.001, aged mice versus young mice. (E, F) Levels of *Nadk* (E) and *Nadk2* (F) transcripts in the islets isolated from the aged and young mice are shown. Data are mean ± SD (*N* = 21). **p < 0.01, ***p < 0.005, aged mice versus young mice. (G) NADP and NADPH contents of islets isolated from the aged and young mice are shown. Data are mean ± SD (*N* = 6). ****p < 0.001, aged mice versus young mice.

To study the difference in glucose tolerance between aged (80‐week‐old) and young (8‐week‐old) male mice, we performed the oral glucose tolerance test (OGTT) with these mice. The area under the curve was 15% lower in the young (8 week) mice than in the aged (80 week) mice, suggesting the young mice had better tolerance than the aged mice (Figure 1A). It was accompanied by changes in insulin secretory capacity. The islets isolated from the young and aged mice were treated under conditions that mimic GSIS. The basal level of insulin released by the aged mouse islets was significantly higher than that of the young mouse islets. However, the level of insulin secreted by the aged mouse islets in response to high glucose was about 40% lower than that of the young ones (Figure 1B). These findings suggest that glucose homeostasis is anomalous in the islets of aged mice. There was a compensatory increase in the weight of aged mouse pancreata (Figure 1C). Their relative weight (% body weight) was about 18% higher than that of young counterparts. Concomitant with this, the fasting plasma insulin level of the aged mice was higher than that of young mice (Figure 1D). The NADPH and NADP levels of aged mouse islets decreased by 28% and 20%, respectively, compared to those of young mouse islets (Figure 1G). It was associated with a reduction in the expression of *Nadk* and *Nadk2* genes in islets. The levels of *Nadk* and *Nadk2* transcripts in the aged mouse islets were lower by 31% and 28%, respectively, than those in young mouse islets (Figure 1E,F). Our findings imply that the expression of *Nadk* and *Nadk2* genes and NADP(H) pool in pancreatic islets diminish with aging.

### 3.2 Expression of *Nadk* and *Nadk2* Genes Plays an Important Regulatory Role in Insulin Secretion

To study if expression of *Nadk* and *Nadk2* genes is causally related to the insulin secretory capacity of β‐cells, we generated the *Nadk* KD, *Nadk2* KD, and NTC cells. Expression of *Nadk* and *Nadk2* genes was specifically suppressed in the respective KD cells. The *Nadk* and *Nadk2* transcript levels were lowered by 55% and 73%, respectively, in *Nadk* KD and *Nadk2* KD cells, as compared to NTC cells (Figure S2A,B). Their capacity to generate NADP(H) was impaired in these cells. The abundance of NADPH and NADP decreased by nearly 30% and 12%, respectively, in these KD cells (Figure S2C). To study if knockdown of *Nadk* and *Nadk2* genes in β‐cells hampers their GSIS response, we collected the culture supernatant from KD and NTC cells after high glucose treatment and determined the secreted insulin. The fold induction of insulin secretion by *Nadk* KD and *Nadk2* KD cells after 1 h glucose stimulation was reduced by over 75% (Figure S2D), suggesting that NADK and NADK2 are essential to the GSIS response.

### 3.3 GSIS‐Associated Changes in β‐Cell Metabolism

**FIGURE 2:**
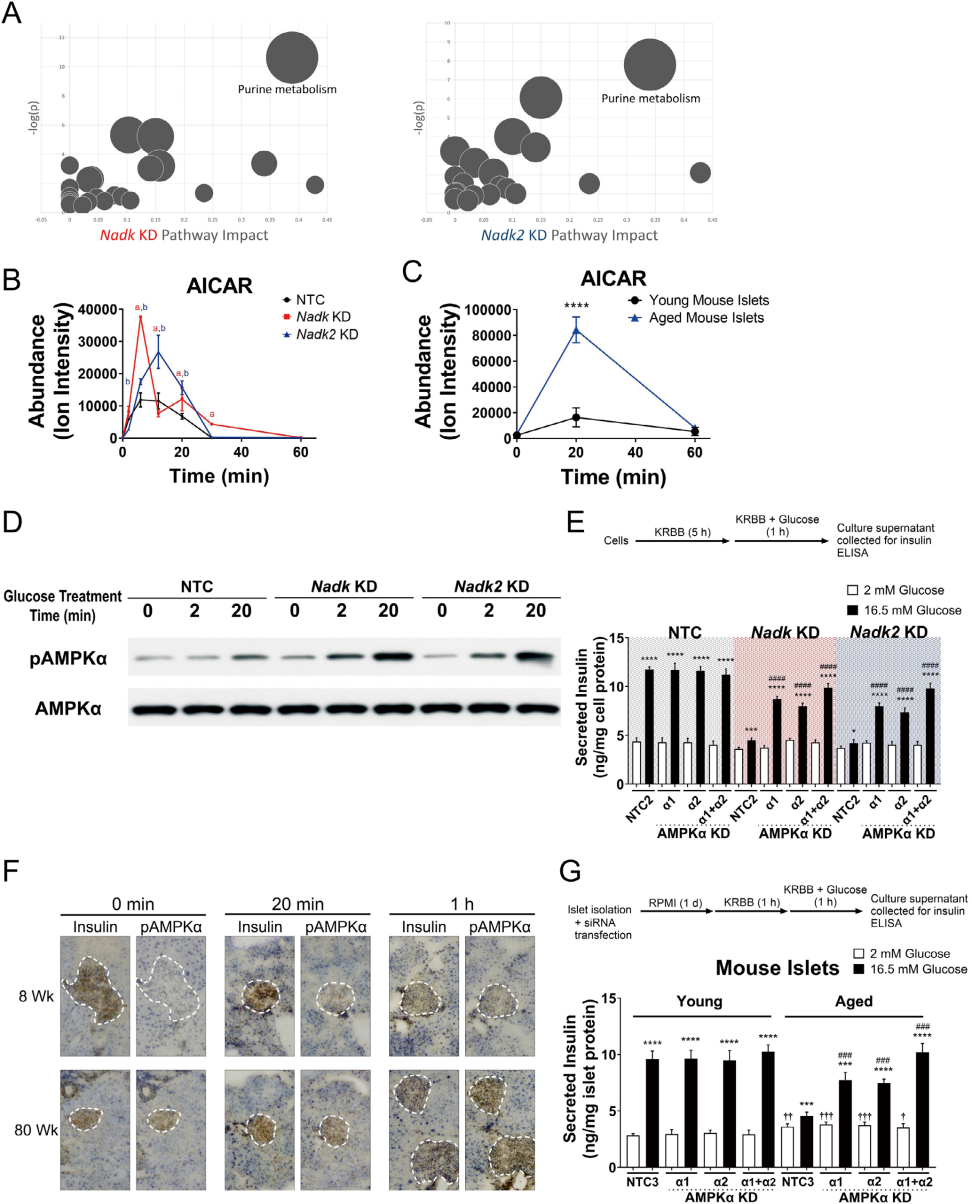
AICAR increases and AMPK is activated to a higher extent in high glucose‐stimulated NADK‐ or NADK2‐deficient cells and in the islets of aged mice after oral glucose challenge. (A) NTC, *Nadk* KD, and *Nadk2* KD cells were treated as described in Figure S2D and were harvested for metabolomic analysis. Pathway view of significant metabolic pathways in *Nadk* KD (left panel) and *Nadk2* KD (right panel) cells versus NTC cells is shown. (B) Levels of AICAR in these cells are shown and given as the intensity of the corresponding ion (*ion intensity*). Data are mean ± SD (*N* = 9). ^a^ *p* < 0.05, *Nadk* KD versus NTC cells; ^b^ *p* < 0.05, *Nadk2* KD versus NTC cells. (C) AICAR levels in the isolated islets of young and aged mice which were treated with 16.5 mM glucose for the indicated times are shown and given as the intensity of the corresponding ion (*ion intensity*). Data are mean ± SD (*N* = 6). ****p < 0.001, aged versus young mouse islets. (D) Immunoblots of phosphorylated AMPKα (*pAMPKα*) and total AMPK (*AMPKα*) in NTC, *Nadk* KD, and *Nadk2* KD cells which were stimulated with 16.5 mM glucose for the indicated times are shown. A representative experiment out of three is shown. (E) NTC, *Nadk* KD, and *Nadk2* KD cells were further transduced with the lentiviral vector(s) encoding NTC2, *Prkaa1* (*AMPKα KD α1*)‐ or *Prkaa2* (*AMPKα KD α2*)‐specific shRNA(s), or both (*AMPKα KD α1 + α2*), respectively. The resultant cells were treated as described in the experimental scheme (top panel), and the insulin levels of culture supernatants are shown. Data are mean ± SD (*N* = 12). *p < 0.05, ***p < 0.005, ****p < 0.001, 16.5 versus 2 mM glucose treatment; ^####^ *p* < 0.001, AMPKα KD versus the NTC2‐expressing cells receiving the 16.5 mM glucose treatment. (F) Representative images show IHC staining of pAMPKα and insulin in the islets of the aged and young mice which were fed with a bolus of glucose solution for the indicated times (original magnification: 100×). (G) Aged and young mouse islets were transfected with NTC3, or the siRNA(s) against *Prkaa1* (*AMPKα KD α1*), *Prkaa2* (*AMPKα KD α2*) or both genes (*AMPKα KD α1 + α2*), respectively. The resultant islets were treated as described in the experimental scheme (top panel), and the insulin levels of culture supernatants are shown. Data are mean ± SD (*N* = 6). ***p < 0.005, ****p < 0.001, 16.5 versus 2 mM glucose treatment; ^###^ *p* < 0.005, AMPKα KD versus NTC3‐transfected islets receiving the 16.5 mM glucose treatment; ^†^ *p* < 0.05, ^††^ *p* < 0.01, ^†††^ *p* < 0.005, versus NTC3‐transfected young mouse islets treated with 2 mM glucose.

To study the changes in metabolism and/or metabolic signaling accompanying the GSIS response, we apply a global metabolomic approach to analyze the *Nadk* KD, *Nadk2* KD, and NTC cells at different time points after high glucose treatment. The data were subjected to orthogonal partial least squares‐discriminant analysis (OPLS‐DA) and Metaboanalyst 5.0 analyses. The metabolic pathway analysis pointed to purine metabolism as the significantly affected pathway in the KD cells (Figure 2A). A number of metabolites involved in purine metabolism are shown in Figure S3. There were decreases in levels of such metabolites as IMP, GMP, and AMP after high glucose stimulation. In contrast, the cellular 5‐aminoimidazole‐4‐carboxamide ribonucleotide (AICAR) level was elevated in NTC cells as early as 2 min post‐glucose stimulation, peaked at 12 min, and declined afterwards. However, for the *Nadk2* KD cells, the AICAR peak level was 2.3‐fold that of NTC cells. The AICAR level in *Nadk* KD cells reached its zenith at 6 min and was 3.2‐fold that of NTC cells (Figure 2B). Such findings advocate that NADK and NADK2 are important in the regulation of AICAR production. This also raises the possibility that the age‐dependent decreases in *Nadk* and *Nadk2* transcript levels may be causally related to impaired insulin secretion. To test such possibility, we isolated mouse pancreatic islets for high glucose treatment that mimicked the GSIS and determined the abundance of AICAR in islets. As shown in Figure 2C, the AICAR level increased in islets after glucose stimulation. Still, the peak AICAR level in aged mouse islets was 5 times that of young mouse islets. These findings suggest that the reduction in *Nadk* and *Nadk2* gene expression is associated with AICAR accumulation during the GSIS response.

### 3.4 AMPK Plays Regulatory Roles in GSIS

It is known that AICAR activates AMP‐activated protein kinase α (AMPKα), whose activation negatively regulates insulin secretion (Leclerc et al. 2004; Tsuboi et al. 2003). It is possible that the excessive AMPK activation in KD cells is inhibitory to insulin secretion. The *Nadk* KD, *Nadk2* KD, and NTC cells were stimulated with high glucose and harvested at different time points for examination of phosphorylated AMPKα (pAMPKα). The pAMPKα level increased modestly in NTC cells within the initial 20 min of high glucose stimulation. Nonetheless, the AMPKα phosphorylation was significantly elevated in *Nadk* KD and *Nadk2* KD cells (Figure 2D). We examined whether the knockdown of AMPKα gene (*Prkaa1* and *Prkaa2*) affected their insulin secretory capacity. The shRNA against *Prkaa1*, *Prkaa2*, or both effectively suppressed the expression of the corresponding genes (Figure S4A). Knockdown of *Prkaa1* and/or *Prkaa2* gene expression largely restored the GSIS response in *Nadk* KD and *Nadk2* KD cells (Figure 2E). Likewise, treatment of the *Nadk* KD and *Nadk2* KD cells with the AMPK inhibitor BAY‐3827 (Jia et al. 2024) or compound C augmented their insulin secretion in response to 16.5 mM glucose treatment (Figure S5B,E). Our findings suggest that AMPK plays regulatory roles in GSIS response.

### 3.5 Differential Activation of AMPK in the Aged and Young Mouse Islets Impairs Their GSIS Response

It is possible that the aging‐associated decline in NADK and NADK2 expression may contribute to AMPK phosphorylation in islets and a reduction in their GSIS response. To study such a possibility, we isolated the islets from the young and aged mice, which had been fasted for 6 h and administered glucose orally. The pancreata were collected and processed for immunohistochemical examination of pAMPKα. The pAMPKα level increased in the aged mouse islets, as compared to that of young mouse islets (Figure 2F, Figure S6). Transfection of mouse islets with the siRNAs against *Prkaa1* and/or *Prkaa2* genes specifically reduced the expression of the corresponding genes (Figure S4B). Knockdown of *Prkaa1* and/or *Prkaa2* gene expression in the aged mouse islets effectively elevated their insulin secretory response (Figure 2G). A similar enhancement of the GSIS response was found in the aged mouse islets treated with BAY‐3827 or compound C (Figure S5C,F). These findings suggest that increased AMPK activation in the aged mouse islets contributes to their impaired GSIS response.

### 3.6 Generation of AICAR From IMP in the *Nadk*‐Deficient Cells Upon High Glucose Stimulation

**FIGURE 3:**
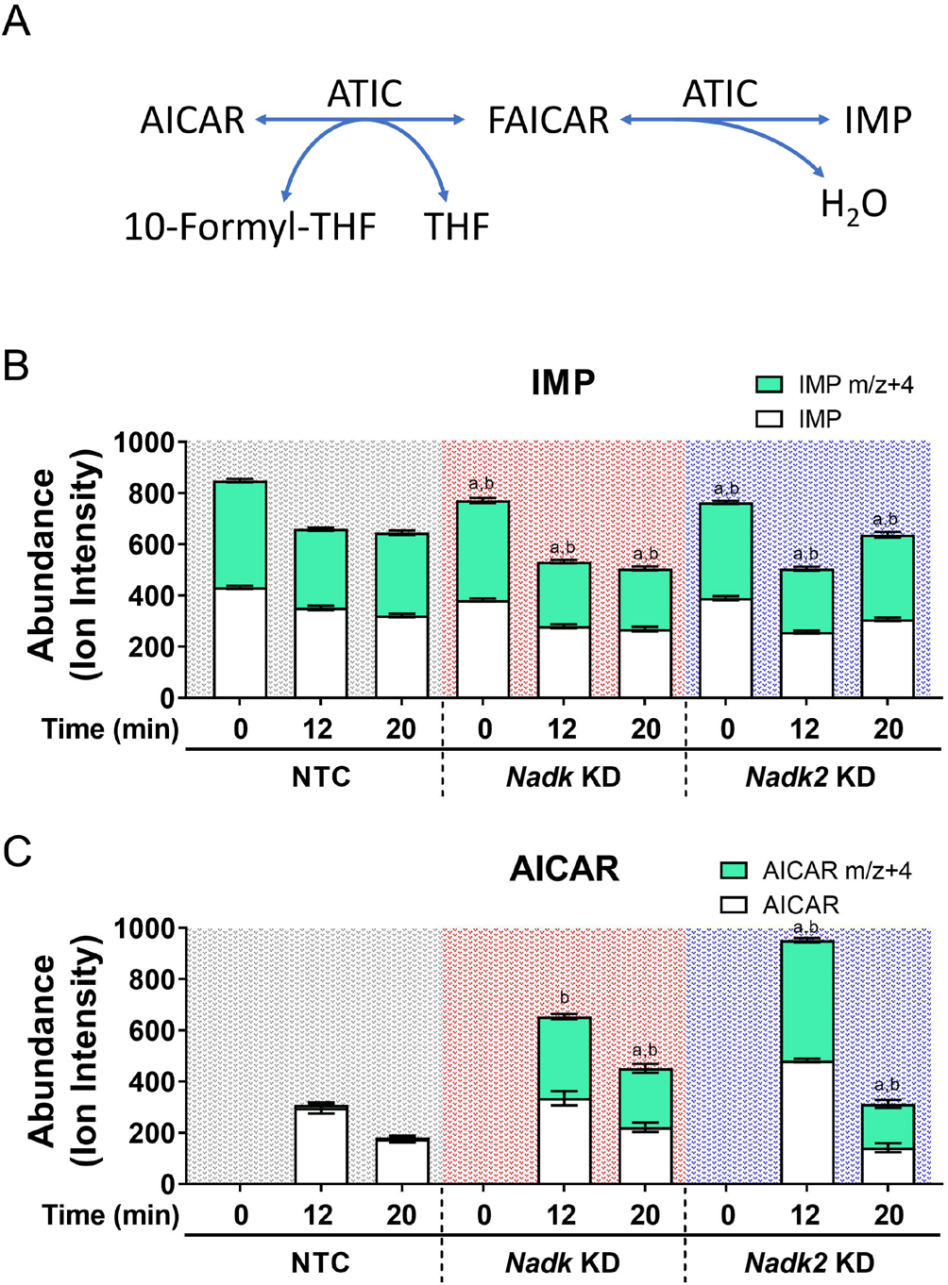
Generation of AICAR from IMP in β‐cells during GSIS. The NTC, *Nadk* KD and *Nadk2* KD cells were labeled with 80 μM [^15^N_4_]‐inosine, incubated under the high glucose condition for the indicated times, and extracted for analysis of IMP and AICAR. (A) Simplified diagram shows the ATIC‐mediated conversion between AICAR and IMP (KEGG reaction identifier; R01127 and R04560). The abundance of IMP (white bar) and IMP (m/z + 4) (green bar) (B), and AICAR (white bar) and AICAR (m/z + 4) (green bar) (C) are expressed as the ion intensities of corresponding ions. Data are mean ± SD (*N* = 6). ^a^ *p* < 0.05, unlabeled metabolite in KD cells versus that of NTC cells; ^b^ *p* < 0.05, labeled (m/z + 4) metabolite in KD cells versus that of NTC cells.

IMP is normally formed from AICAR in a forward reaction catalyzed by aminoimidazole ribonucleotide transformylase/inosine monophosphate cyclohydrolase (ATIC) (Figure 3A). To study how AICAR is formed during the GSIS response of β‐cells, we labeled the *Nadk* KD, *Nadk2* KD, and NTC cells with [^15^N_4_]‐inosine, treated the labeled cells, and tracked the formation of cellular AICAR after high glucose treatment. The [^15^N_4_]‐inosine can be converted to [^15^N_4_]‐IMP. For both KD and NTC cells, substantial conversion of [^15^N_4_]‐inosine to [^15^N_4_]‐IMP occurred (Figure 3B). Intriguingly, AICAR that was transiently formed in NTC cells during the GSIS response was mainly the unlabeled one. The amount of [^15^N_4_]‐AICAR formed from [^15^N_4_]‐IMP was minimal. However, during high glucose stimulation of KD cells, the unlabeled and [^15^N_4_]‐AICAR were produced in appreciable amounts (Figure 3C). This suggests that the unlabeled AICAR that accumulated in NTC cells during the GSIS response is probably derived from the de novo purine biosynthesis. In contrast, the labeled AICAR that increased significantly in high glucose‐treated KD cells can only be formed from the labeled IMP. It is likely that the decline in *Nadk* and *Nadk2* gene expression in the latter cells promotes the formation of AICAR from IMP.

**FIGURE 4:**
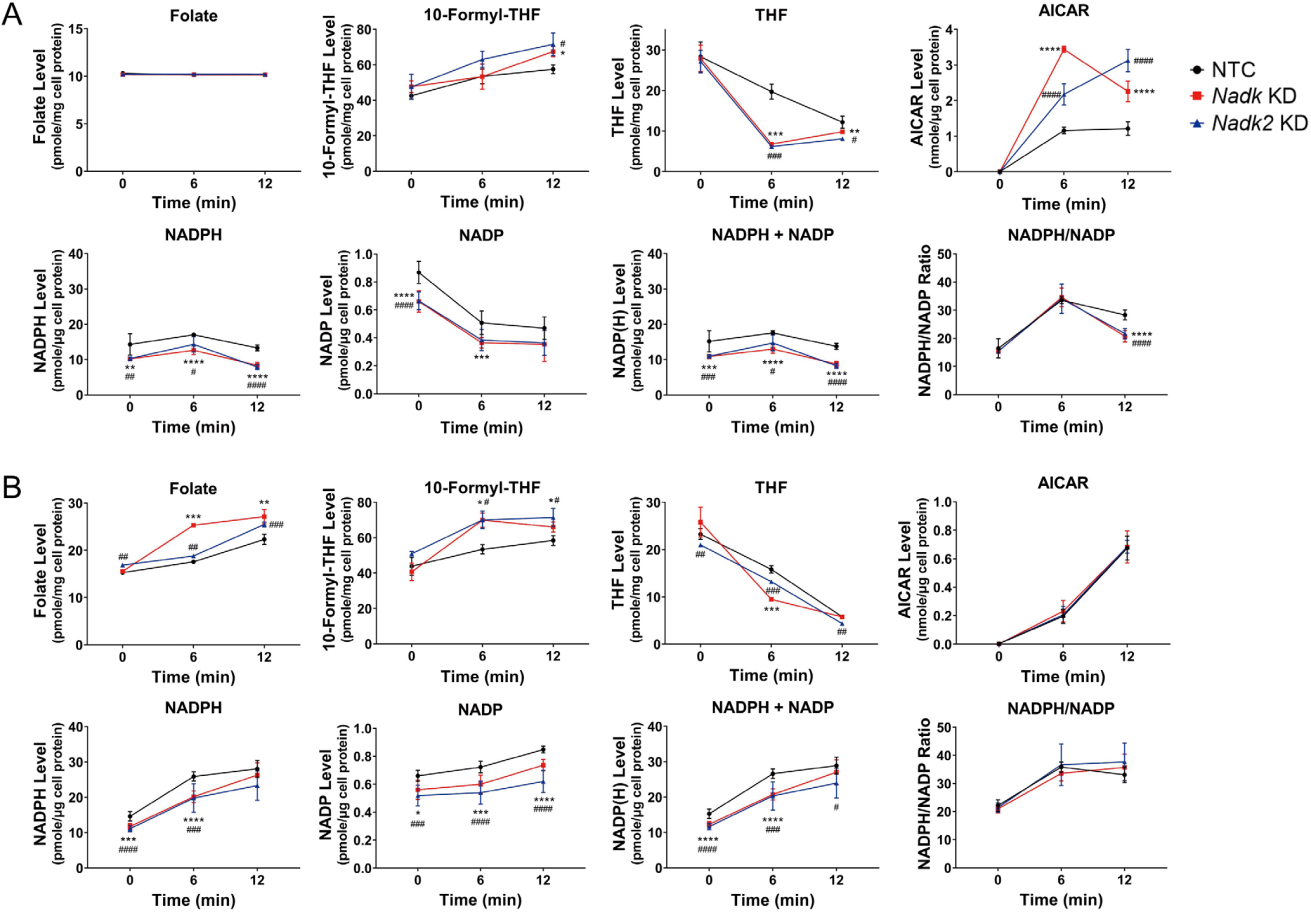
Alteration of the folate and NADP(H) metabolism in the high‐glucose‐stimulated NADK‐ or NADK2‐deficient cells. The NTC, *Nadk* KD, and *Nadk2* KD cells were treated without (A) or with (B) 3 μM folate, stimulated with 16.5 mM glucose for the indicated times, and harvested for analysis of folate, 10‐formyl‐THF, THF, NADP, NADPH, and AICAR. Total NADP(H) (i.e., NADP +NADPH) and NADPH/NADP ratio were calculated accordingly. (KEGG pathway identifier for the NAD(H) and NADP(H) metabolism: hsa00760; KEGG pathway identifier for the folate cycle: hsa00670) The metabolite level is expressed as pmole/mg, pmole/μg or nmole/μg cell protein. Data are mean ± SD (*N* = 6). *p < 0.05, **p < 0.01, ***p < 0.005, ****p < 0.001, *Nadk* KD cells versus NTC cells; ^#^ *p* < 0.05, ^##^ *p* < 0.01, ^###^ *p* < 0.005, ^####^ *p* < 0.001, *Nadk2* KD cells versus NTC cells.

10‐Formyltetrahydrofolate (10‐formyl‐THF) is needed to provide a formyl group to AICAR for its transformation to the intermediate 5‐formylaminoimidazole‐4‐carboxamide ribonucleotide (FAICAR). THF formed in the reaction is recycled to 10‐formyl‐THF in a NADPH‐dependent manner. It is plausible that a decrease in NADK or NADK2 expression, and hence the NADP(H) pool abatement, affects the rate of THF/10‐formyl‐THF recycling. This may favor the formation of AICAR from IMP. Consistent with such a notion, there were differences in 10‐formyl‐THF and THF between KD and NTC cells stimulated with high glucose. The THF levels in *Nadk* KD and *Nadk2* KD cells were 66% and 69% lower than those of NTC cells, respectively, at 6 min post‐treatment (Figure 4A, *THF* panel). The 10‐formyl‐THF levels increased by 18% and 25% in *Nadk* KD and *Nadk2* KD cells at 12 min post‐treatment, respectively (Figure 4A, *10‐Formyl THF* panel). These findings are consistent with the occurrence of the reverse transformylase‐catalyzed reaction that produces 10‐formyl‐THF at the consumption of THF in these cells.

The changes in folate metabolism during glucose stimulation are associated with changes in NADP(H) metabolism. The NADPH level in NTC cells increased mildly by 20% at 6 min post‐treatment and returned to the original level at 12 min post‐treatment. In contrast, the NADP level decreased by over 40% at 6 min post‐treatment and remained unchanged. The NADPH and NADP levels in *Nadk* KD and *Nadk2* KD cells exhibited a similar trend of changes (Figure 4A, *NADPH* and *NADP* panels). The NADPH/NADP ratio of NTC cells increased 2‐fold at 6 min post‐treatment and declined mildly thereafter. For *Nadk* KD and *Nadk2* KD cells, the NADPH/NADP ratios were significantly lowered at 12 min post‐treatment (Figure 4A, *NADPH/NADP* panel).

We further tested if supplementation of these *Nadk* KD, *Nadk2* KD, and NTC cells with 3 μM folate can rescue their GSIS response. During the course of high glucose treatment, the folate supplementation increased cellular folate levels in KD and NTC cells (Figure 4B, *Folate* panel). The THF levels were 35% and 15% lower in *Nadk* KD and *Nadk2* KD cells than in NTC cells at 6 min post‐treatment, respectively (Figure 4B, *THF* panel). Concomitant with these changes, the 10‐formyl‐THF levels in *Nadk* KD cells increased by 30% and 13% at 6 and 12 min post‐treatment. Those of *Nadk2* KD cells were elevated by 31% and 22% at the same time points (Figure 4B, *10‐Formyl THF* panel). In the folate‐supplemented NTC and KD cells, the levels of NADPH and NADP increased substantially with treatment time (Figure 4B, *NADPH* and *NADP* panels). The NADPH/NADP ratios of NTC and KD cells were around 30% higher than those of the untreated counterparts. This parameter increased and remained steady at 12 min post‐treatment (Figure 4B, *NADPH/NADP* panel). Intriguingly, the AICAR levels in NTC and KD cells were about 20% and 56% of those of untreated NTC cells at 6 and 12 min post‐treatment, respectively (Figure 4A vs. 4B, *AICAR* panel), suggesting that folate treatment effectively suppresses the AICAR production during GSIS.

### 3.7 Folate Supplementation Enhances the Insulin Secretory Capacity of β‐Cells and Islets

**FIGURE 5:**
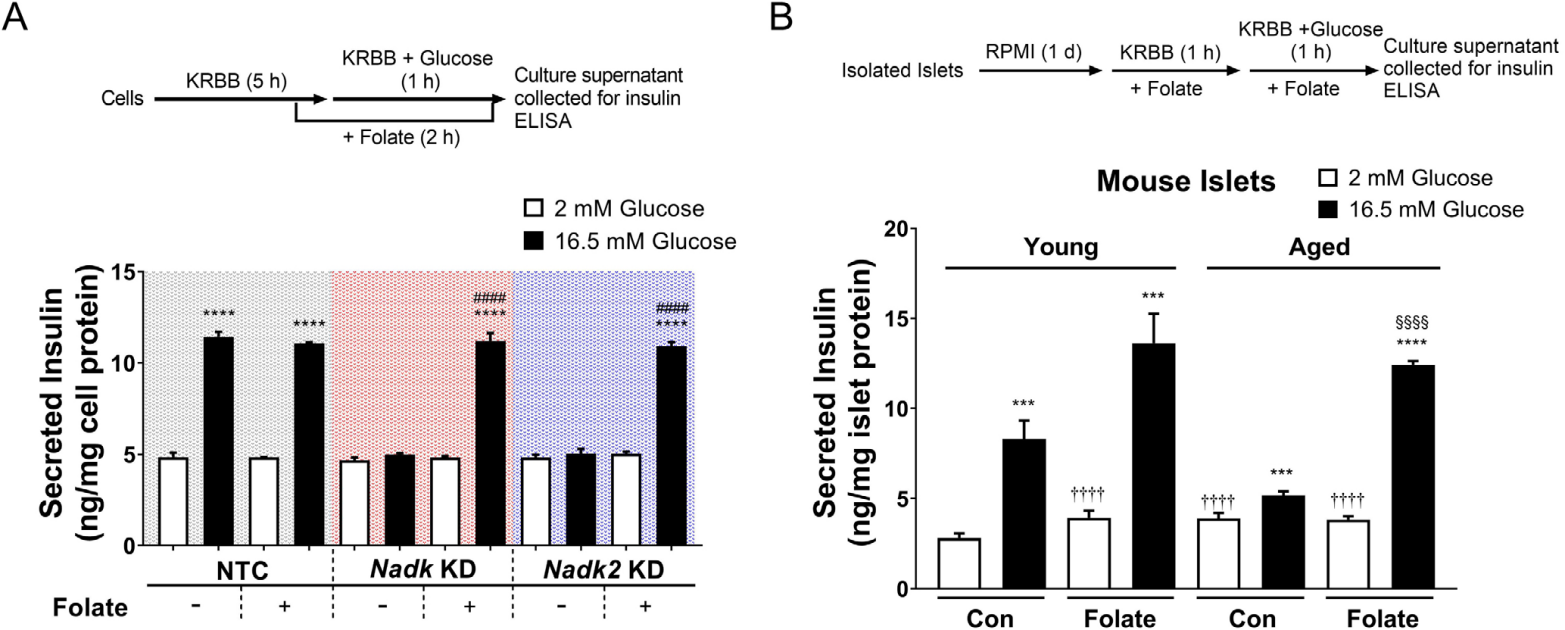
Folate supplementation restores the insulin secretory capacity of *Nadk* KD, *Nadk2* KD cells, and the aged mouse islets. (A) NTC, *Nadk* KD, and *Nadk2* KD cells were treated as described in the experimental scheme (top panel). The insulin levels of culture supernatants are shown. Data are mean ± SD (*N* = 6). ****p < 0.001, 16.5 versus 2 mM glucose treatment; ^####^ *p* < 0.001, folate‐treated versus untreated cells receiving the 16.5 mM glucose treatment. (B) Islets isolated from the aged and young mice were treated without (*Con*) or with (*Folate*) 3 μM folate and stimulated with 16.5 mM glucose as described in the experimental scheme (top panel). The insulin levels of culture supernatants are shown. Data are mean ± SD (*N* = 6). ***p < 0.005, ****p < 0.001, 16.5 versus 2 mM glucose treatment; ^††††^ *p* < 0.001, versus young mouse islets treated with 2 mM glucose but without folate; ^§§§§^ *p* < 0.001, folate‐treated versus untreated aged mouse islets receiving the 16.5 mM glucose treatment.

The changes in the folate and NADP(H) metabolism may lead to an altered GSIS response of these cells. Folate supplementation did not affect the basal insulin secretion by *Nadk* KD and *Nadk2* KD cells. However, it did completely restore the levels of insulin secreted by high glucose‐stimulated KD cells to those attained by the similarly treated NTC cells (Figure 5A). These findings suggest that the changes in GSIS response of either *Nadk* KD or *Nadk2* KD cells may be attributed to an altered folate metabolism.

The findings about the folate supplementation of KD and NTC cells prompt us to examine whether the same treatment may improve the insulin secretory capacity of mouse islets. The young and aged mouse islets were isolated and treated for analysis of GSIS response. Folate supplementation significantly potentiated the insulin secretion by high glucose‐stimulated young and aged mouse islets (Figure 5B). The insulin secretion by high glucose‐stimulated aged mouse islets was restored to the level that was 1.5‐fold that attained by the untreated high glucose‐stimulated young mouse islets. These findings suggest that the aging‐associated reduction in insulin secretory capacity may be related to altered *Nadk* and *Nadk2* gene expression and abnormal folate metabolism. Folate supplementation can augment the GSIS response of the aged mouse islets.

## 4 Discussion

We demonstrate that the islets of aged mice expressed less *Nadk* and *Nadk2* transcripts and have less NADP(H) content than their young counterparts, which correlated with their diminished GSIS response. Knockdown of *Nadk* and *Nadk2* gene expression in β‐cells led to reduced GSIS and changes in purine metabolism. AICAR accumulated to higher levels in KD cells and the aged mouse islets, and caused more robust AMPK activation. AMPK activation was inhibitory to GSIS response, as revealed by the ability of AMPK‐specific shRNAs/siRNAs and AMPK inhibitors to promote the insulin secretory capacity of KD cells and the aged mouse islets. Stable isotope labeling study demonstrated that an excessive amount of AICAR was formed from IMP in KD cells during the course of GSIS. The enhanced AICAR formation was accompanied by changes in folate metabolism. It is probable that the insufficiency of NADP(H) supply affects the folate metabolism, which may be instrumental in determining the directionality of ATIC transformylase‐catalyzed reaction. Folate supplementation enhanced the high glucose‐stimulated insulin secretion by KD cells and the aged mouse islets. Our findings suggest that decreases in *Nadk* and *Nadk2* gene expression in the aged mouse islets are causally related to diminished GSIS response.

The canonical model of GSIS entails coupling of the nutrient metabolism, mostly that of glucose, to the insulin secretory process via a myriad of MCFs. These MCFs include regulatory MCFs (e.g., adenine nucleotide, malonyl‐coenzyme A, etc.) that regulate the metabolic networks and effectory MCFs (e.g., ATP, cAMP, NADP(H), etc.) that are directly involved in signal amplification and triggering exocytosis (Prentki et al. 2013). Interaction and balance between different pathways are essential to the proper functioning of the entire metabolic signaling process. Previous studies demonstrated that NADP(H) is required for the redox cycling of glutaredoxin, which in turn redox‐regulates other exocytosis proteins (Ivarsson et al. 2005). The Kv channel that is required for the repolarization of β‐cell membrane potential contains an NADPH molecule as a cofactor (Jitrapakdee et al. 2010). Oxidation of the bound cofactor inhibits channel inactivation (Pan et al. 2011). A new model of metabolic and redox oscillations involved in β‐cell metabolic sensing and signaling, known as the MitoCat‐MitoOx model, has been recently proposed (Merrins et al. 2022). Cellular redox status and mitochondrial activity change periodically in the regulation of insulin exocytosis. During the Mito_Cat_ phase, elevation of mitochondrial NADH leads to high ROS production, and the pyruvate‐malate shuttle‐mediated efflux of malate from mitochondria to cytosol promotes ROS formation through the actions of malic enzyme and NADPH oxidase 4. During the Mito_Ox_ phase, NADPH is generated via the isocitrate dehydrogenase 1‐mediated reaction from isocitrate, which is exported from mitochondria through the oxoglutarate carrier/citrate–isocitrate carrier. NADPH suppresses exocytosis through the redox regulation of exocytosis effector proteins and helps to reset the metabolic cycles involved in insulin secretion. Our present findings suggest that NADP(H) may have an alternative biochemical role in β‐cells. Insufficiency of NADK and NADK2 activities and NADP(H) supply may alter folate metabolism and other metabolic pathways, such as purine metabolism. Consistent with this notion, the NADK inhibitor thionicotinamide adenine dinucleotide phosphate dramatically reduces the NADP(H) pool (Tedeschi et al. 2015) and causes the degradation of dihydrofolate reductase (Hsieh et al. 2013). This impinges on folate cycling and the biosynthesis of purine, thymidylate, and other metabolites, leading to cell growth inhibition. A similar molecular mechanism may be operational in β‐cells. Coupling of the redox metabolism of NADP(H) and the folate cycle may be regulatory to reactions involved in purine biosynthesis, for instance, the ATIC‐catalyzed reactions. In the normal β‐cells, the flux of the upstream purine biosynthesis pathway may be altered upon glucose stimulation. However, the flux of the forward reactions catalyzed by ATIC may not change proportionately or may even decrease (Figure 3), leading to an initial accumulation of AICAR and 10‐formyl‐THF and a decline in the IMP level. Different folate derivatives are interconverted through a series of reactions that constitute the folate cycle or one‐carbon cycle, in which serine, ATP (ADP) and NADP(H) are involved (Tedeschi et al. 2013; Zheng and Cantley 2019). Previous findings indicate that the folate cycling contributes to the NADPH pool of INS1E β‐cells after activation by glibenclamide, an ATP‐sensitive potassium channel (K_ATP_) inhibitor (Bui et al. 2023). It is possible that the interconvertible reactions of folate derivatives, including 10‐formyl‐THF, THF, 5,10‐methylene‐THF, and 5,10‐methenyl‐THF, can be diverted to NADPH production during GSIS, particularly in the case of NADP(H) insufficiency. It follows that the pool size and redox state of NADP(H) may alter the fluxes of various NADP(H)‐dependent reactions of folate derivatives, for example, the aldehyde dehydrogenase 1 family member L2 (ALDH1L2)‐catalyzed interconversion between 10‐formyl‐THF and THF. This, in turn, affects the folate‐dependent metabolic reactions, for example, the ATIC‐catalyzed reaction. Dwindling of NADP(H) pool, as what was observed with the glucose‐stimulated *Nadk* KD or *Nadk2* KD cells, may reprogram the folate‐dependent metabolic reaction to furnish sufficient NADPH for the maintenance of cellular redox status and/or anabolism, and for driving the redox/metabolic cycles involved in insulin secretion. This may account for the formation of AICAR and 10‐formyl‐THF from IMP and THF (in the reverse reaction catalyzed by ATIC). 10‐Formyl‐THF can be converted to THF to generate NADPH by ALDH1L2, which is abundant in pancreatic islets (Krupenko et al. 2010; Pelligra et al. 2023). Coupling of the ALDH1L2‐catalyzed forward reaction and the ATIC‐catalyzed reverse reaction may occur to a significant extent in the high glucose‐stimulated *Nadk* KD or *Nadk2* KD cells. This contributes to the NADPH pool to meet the metabolic need of these cells during GSIS. It is consistent with our finding that folate supplementation increases the NADPH/NADP ratio of β‐cells before glucose stimulation and counters the GSIS‐induced decline in the NADPH/NADP ratio. This is associated with the mitigation of AICAR accumulation (Figure 4). The intimate interaction between NADP(H) metabolism and the folate cycle is also reflected by the increases in the NADP and NADPH pools in the folate‐supplemented β‐cells (Figure 4). Fan et al. reported that NADPH production is dependent on folate (Fan et al. 2014). A similar mechanism may be operational in the folate‐supplemented β‐cells. Our findings imply that NADP(H) metabolism and the folate cycle interact to modulate purine metabolism during the GSIS response of β‐cells. Our findings are in agreement with the findings of clinical studies and trials. Insulin‐resistant patients were found to have lower folate levels (Tavares Bello and Duarte 2021). Folate supplementation improves glycemic control in human cohorts (Asbaghi et al. 2021; Li et al. 2017; Lind et al. 2019).

It is unlikely that the formation of [^15^N_4_]‐AICAR in glucose‐stimulated KD cells could originate from degradation of [^15^N_4_]‐inosine and inclusion of all four [^15^N] atoms in a newly synthesized 5‐aminoimidazole‐4‐carboxamide ring of AICAR. The nitrogen of purine is derived from glutamine, glycine, and aspartate during its de novo synthesis. The probability that all four nitrogen atoms in the aminoimidazole‐4‐carboxamide ring are labeled in this way is very low. Moreover, a relatively high level of intact [^15^N_4_]‐IMP was present in NTC and KD cells before GSIS. If substantial [^15^N_4_]‐inosine degradation and transformation had occurred, a high level of [^15^N_4_]‐AICAR would have been present in glucose‐stimulated NTC cells. In fact, we did not detect any [^15^N]‐ (m/z + 1), [^15^N_2_]‐ (m/z + 2) and [^15^N_3_]‐containing (m/z + 3) isotopologues of AICAR in NTC or KD cells, ruling out the possibility that [^15^N_4_]‐AICAR arises from degradation of [^15^N_4_]‐inosine and de novo biosynthesis.

The last two steps of the purine de novo synthesis are catalyzed by ATIC. ATIC transfers the formyl group from 10‐formyl‐THF to AICAR to form 5‐formamidoimidazole‐4‐carboxamide ribotide (FAICAR). The latter is subsequently converted to IMP through the IMP cyclohydrolase activity of ATIC (Pareek et al. 2021). Both activities reside on the same polypeptide. The transformylase reaction is reversible, with the rate of the reverse reaction being 2–3 fold higher than the forward reaction rate (Bulock et al. 2002). The cyclohydrolase reaction is largely unidirectional in the direction of IMP formation, driving the bifunctional reaction forward. The mechanism underlying the reverse formation of AICAR from IMP is currently unknown. One possibility is that ATIC might catalyze both reactions in a reverse manner by itself. Alternatively, other hitherto unknown IMP 1,2‐hydrolases (ring‐opening) or cyclohydrolases may catalyze the ring‐opening reaction to form FAICAR, which can be subsequently converted to AICAR.

The NAD kinases may play an important role in β‐cell physiology. Gray et al. demonstrated that overexpression of the *Nadk* gene, which increased the NADPH pool and NADPH/NADP ratio, could augment insulin secretion by INS‐1832/13 cells upon glucose stimulation. The effect of *Nadk* gene knockdown on them was opposite (Gray et al. 2012). However, the underlying mechanism remained elusive. Kim et al. reported that the *Nadk2*‐knockout mice exhibited increased hepatic insulin resistance and reduced glucose tolerance (Kim et al. 2022), suggesting its role in metabolic homeostasis. Our present findings indicate that NADK2 plays a role in GSIS response, expanding its role in metabolic homeostasis. The age‐dependent decline in *Nadk* and *Nadk2* expression in islets is associated with a decrease in NADP(H) content and GSIS level (Figure 1). Though the basal secretion of the aged mouse islets was higher than that of young ones, their glucose‐stimulated secretory capacity decreased significantly. The total mass of the pancreas of aged mice was elevated, as compared to that of young ones, probably reflecting a compensatory hypertrophy of islets during aging. These findings are consistent with previous observations (Leiter et al. 1988; Montanya et al. 2000; Rankin and Kushner 2009). It is intriguing to study whether the *NADK* and *NADK2* genes are underexpressed in the aged human islets. We analyzed a transcriptomic dataset of the human islet tissues collected at different ages (NCBI Gene Expression Omnibus accession number: GSE165121) (Seiron et al. 2021). The *NADK* and *NADK2* transcript levels are lower, albeit non‐significantly, in the individuals aged above 40 than in those aged below 40 (our unpublished data). Moreover, analysis of a single‐cell RNA‐seq dataset of human islets, which were harvested from individuals with and without type 2 diabetes (NCBI Gene Expression Omnibus accession number: GSE153855) revealed that the expression of the *NADK* and *NADK2* genes appears to be lower in the islets of diabetic individuals (Ngara and Wierup 2022) (our unpublished data). These findings advocate that the age‐dependent changes in *NADK*/*Nadk* and *NADK2*/*Nadk2* expression may be related to the age‐dependent changes in insulin secretory capacity and susceptibility to diabetes.

As mentioned in the preceding paragraph, folate metabolism plays an important role in β‐cell metabolic signaling and physiology. A recent study has shown that activating transcription factor 4 (ATF4) is essential to the activation of genes encoding enzymes involved in the serine‐related mitochondrial folate metabolism. A genetic manipulation study has shown that ATF4 upregulates the expression of these genes and reduces the GSIS response (Pelligra et al. 2023). It is plausible that the shifting of glycolysis toward serine‐linked mitochondrial folate metabolism alters levels of NADP(H) and various folate derivatives, and affects the GSIS. In addition, Bui et al. showed that the dihydrofolate reductase inhibitor methotrexate suppresses NADP reduction in glucose‐stimulated β‐cells (Bui et al. 2023). These findings are consistent with the importance of the folate cycle and NADP(H) in the regulation of insulin secretion.

Purine metabolites are implicated in the regulation of GSIS. ATP is believed to close the K_ATP_ channel and to trigger membrane depolarization of β‐cells, and to sustain ion pumping and exocytosis (Merrins et al. 2022). Adenylosuccinate (S‐AMP), generated from IMP by adenylosuccinate synthase, acts as an insulin secretagogue to stimulate insulin granule exocytosis (Gooding et al. 2015). The present study demonstrated that the AICAR level is transiently elevated in β‐cells during GSIS. The glucose‐induced increase in AICAR (ZMP) was reported with another β cell line, INS‐1 832/13 (Lorenz et al. 2013). The level of this metabolite is higher in KD cells than in NTC cells. This corresponds with a reduction in the amount of insulin secreted upon high glucose stimulation, suggesting an inhibitory role for AICAR in GSIS. It is consistent with the previous finding that AICAR‐induced AMPK activation blocks GSIS in MIN6 cells and isolated rat islets (da Silva Xavier et al. 2003). There have been some controversies about the role of AMPK in insulin secretion. Other studies reported that AMPK activation potentiates insulin secretion by rat and human islets (Akkan and Malaisse 1994; Del Guerra et al. 2005). It is probable that the duration and/or extent of AMPK activation may determine whether it stimulates or suppresses insulin secretion. It may even play different roles during different GSIS phases. Nguyen‐Tu et al. reported that acute AMPK activation is stimulatory to GSIS, while its prolonged activation is inhibitory (Nguyen‐Tu et al. 2022). Such a notion is in agreement with the “two‐way switch” hypothesis in which AMPK activation can either be death‐inducing or cytoprotective, depending on whether the activation level is above or below a certain threshold (Zhao et al. 2022). It is probable that the elevated AMPK activation in KD cells may be inhibitory to insulin secretion, while the modest AMPK activation may be essential to the molecular processes involved in GSIS. The increase in AICAR and AMPK activation in the glucose‐stimulated aged mouse islets is consistent with an inhibitory role of AMPK for the age‐dependent changes in GSIS response.

**FIGURE 6:**
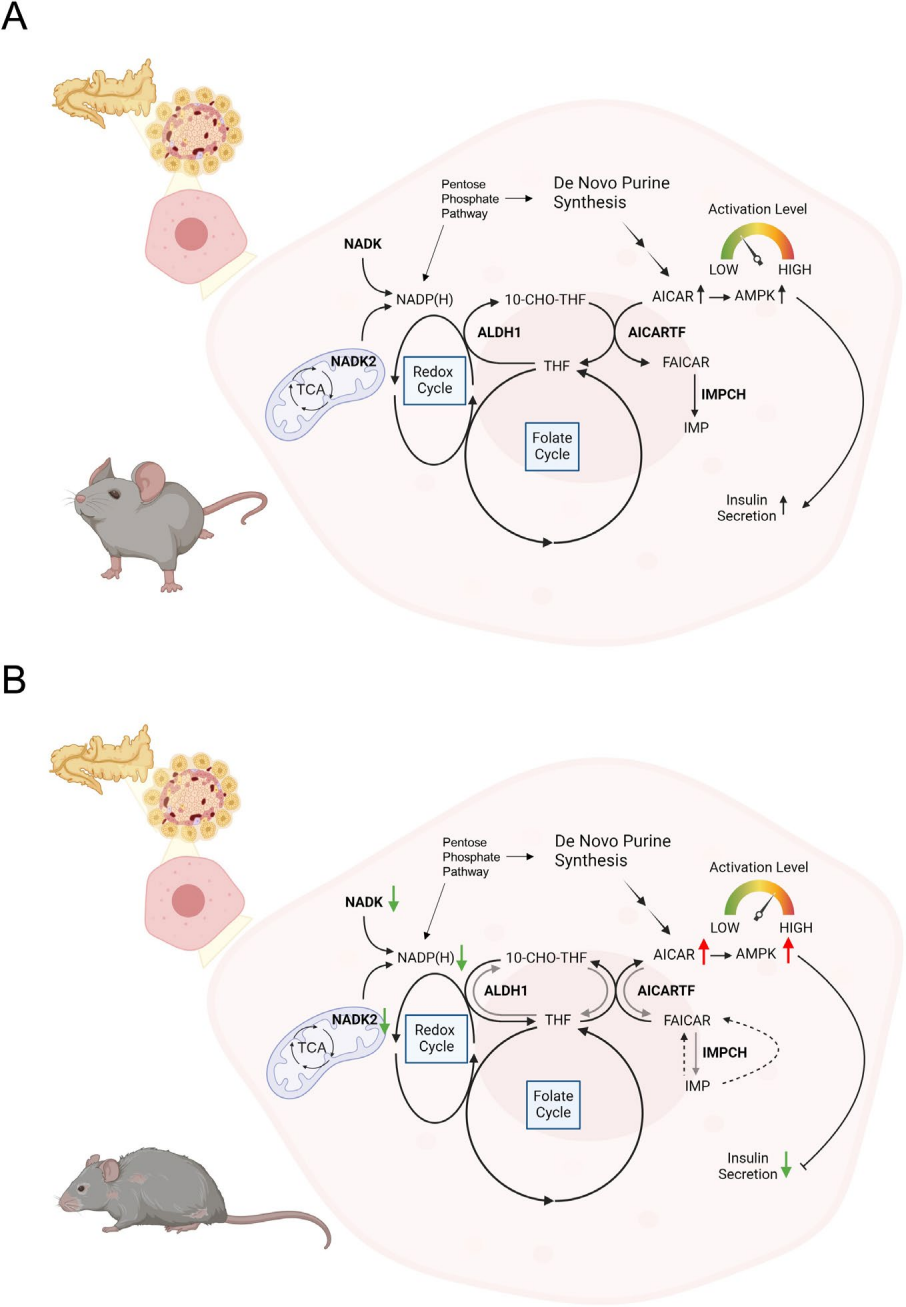
Schematic diagram showing the NADP(H) and folate cycles in regulating GSIS response in the β‐cells of young and aged mouse islets. (A) Normally, in glucose‐stimulated β cells (e.g., those from young mouse islets), the presence of NADK and NADK2 maintain adequate NADP(H) pool and support the interconversion reactions of folate cycle. 10‐Formyl‐THF (10‐CHO‐THF) is needed for AICAR transformylase (AICARTF)‐mediated conversion of AICAR to FAICAR. The THF thus formed may undergo reductive carboxylation to form 10‐CHO‐THF through the ALDH1‐mediated reaction. Alternatively, THF can be converted to other derivatives, such as 5‐methyl‐THF, 5,10‐methylene‐THF and 5,10‐methenyl‐THF via successive reactions in the folate cycle. The reducing equivalents may be transferred to NADP(H), which act as MCF. The GSIS response is accompanied by a transient increase in AICAR that is produced via de novo purine synthesis. A modest increase in AMPK activity (whose intensity is below a certain threshold) is promotional to insulin secretion. (B) However, when β‐cells are deficient in either NADK or NADK2 activity (e.g., in β‐cells of aged mouse islet), the diminished NADP(H) pool may put much burden on the NADP(H)‐dependent metabolic pathways, which is reprogrammed to provide sufficient NADPH for maintenance of cellular redox status and/or anabolism. AICARTF and ALDH1 work coordinately in reverse manner to convert AICAR from FAICAR with generation of NADPH. FAICAR might be formed from IMP via the reverse IMP cyclohydrolase (IMPCH) (dashed straight arrow) or hitherto unknown pathways (dashed curve arrow). Robust AICAR accumulation and AMPK activation (whose intensity exceeds a certain threshold) may be inhibitory to insulin secretion. The green downward arrow indicates a decrease in enzyme activity, expression level or metabolite abundance, while the red upward arrow indicates an increase in such a parameter.

Aging‐associated purine dysregulation occurs in a number of tissues, such as brain, heart, kidney, lung, and pancreas (Li et al. 2021; Zhang, Kerbl‐Knapp, et al. 2021). Changes in folate metabolism and NADP(H) also occur during aging (Bradshaw 2019; Lionaki et al. 2022). Various molecular and cellular processes, including gene regulatory network and signaling, can be affected. It is likely that the age‐related changes in NADP(H), folate, and purine metabolism may contribute to a reduction in the ability of β‐cells to maintain metabolic homeostasis (Figure 6). It cannot be excluded that other metabolic pathways may also be involved. For instance, the pentose phosphate pathway furnishes NADPH and ribose 5‐phosphate. Inhibition of the pentose phosphate pathway is inhibitory to insulin secretion (Spegel et al. 2013). This also raises the possibility of amelioration of glycemic control among the elderly through nutritional supplementation.

## Author Contributions

Conceptualization, H.‐Y.H. and M.‐L.C.; methodology, H.‐Y.H. and M.‐L.C.; software, G.‐J.L. and Y.‐T.L.; validation, G.‐J.L., Y.‐T.L., and M.‐L.C.; formal analysis, G.‐J.L., Y.‐T.L., and M.‐L.C.; investigation, G.‐J.L., Y.‐T.L., M.‐L.C., and H.‐Y.H.; resources, H.‐Y.H., G.L., C.‐Y.C., and M.‐L.C.; data curation, M.‐L.C.; writing – original draft preparation, H.‐Y.H. and M.‐L.C.; writing – review and editing, G.L., C.‐Y.C., H.‐Y.H., and M.‐L.C.; visualization, G.‐J.L., Y.‐H.H., and H.‐Y.H.; supervision, H.‐Y.H.; project administration, H.‐Y.H.; funding acquisition, H.‐Y.H. and M.‐L.C. All authors have read and agreed to the published version of the manuscript.

## Conflicts of Interest

The authors declare no conflicts of interest.

## Supporting information


Data S1.

Data S2.

## Data Availability

The data of the current study are available from the corresponding author upon reasonable request.
