# Supplementary material for: Downregulation of NAD Kinase Expression in β‐Cells Contributes to the Aging‐Associated Decline in Glucose‐Stimulated Insulin Secretion
Source: Aging Cell. 2025 Mar 5;24(4):e70037. doi: 10.1111/acel.70037 (PMC11984695; doi:10.1111/acel.70037)
Supplement: Supplementary file 1 — Data S1. [file ACEL-24-e70037-s001.docx]

**SUPPLEMENTARY MATERIALS**

**SUPPLEMENTAL TABLE**

Table S1. Primers used for quantification of specific gene expression

| Gene | Forward Primer | Reverse Primer |
| --- | --- | --- |
| *Nadk* | 5’-ATGGAGTGATCGTGTCCACC-3’ | 5’-GGCTTCCGGTGACAGCATAA-3’ |
| *Nadk2* | 5’-GGGCTGTAGCAGTGGACAAT-3’ | 5’-TGAATGACCAGGCCTTCGAT-3’ |
| *Prkaa1*^a^ | 5’-GGGAAAGTGAAGGTGGGCAA-3’ | 5’-ATCTCCCGGCGGATTTTTCC-3’ |
| *Prkaa2*^a^ | 5’-CAGGCCATAAAGTGGCAGTTA-3’ | 5’-AAAAGTCTGTCGGAGTGCTGA-3’ |
| *Gapdh*^b^ | 5’-AGGTCGGTGTGAACGGATTTG-3’ | 5’-TGTAGACCATGTAGTTGAGGTCA-3’ |
| *Tubb3*^b^ | 5’-GGCAACTATGTAGGGGACTCAG-3’ | 5’-CCTGGGCACATACTTGTGAG-3’ |

^a^*Prkaa1* is the gene encoding AMPKα1; *Prkaa2* is the gene encoding AMPKα2. ^b^*Gapdh* and *Tubb3* serve, respectively, as normalization controls for quantification of gene transcripts in NIT-1 cells and islets.

**SUPPLEMENTARY FIGURE LEGENDS**

Figure S1. Changes in the body weight (*Body Wt.*, g) of male C57BL/6JNarl mice with their weekly-age. Data are mean ± SD.

Figure S2. The NADK or NADK2 deficiency hampers GSIS response of β-cells. (A, B) The expression levels of *Nadk* (A) and *Nadk2* (B) genes in NTC, *Nadk* KD and *Nadk2* KD cells are shown. Data are mean ± SD (N = 6). ***p < 0.005, vs. NTC cells. (C) The NADP and NADPH contents of NTC, *Nadk* KD and *Nadk2* KD cells are shown. Data are mean ± SD (N = 5). ****p < 0.001, vs. NADPH content of NTC cells; ^§§§^p <0.005, vs. NADP content of NTC cells. (D) The NTC, *Nadk* KD and *Nadk2* KD cells were treated with 16.5 mM glucose. The insulin levels of the culture supernatants collected at various times after glucose stimulation are shown. Data are mean ± SD (N = 6). The p values for comparison between *Nadk* KD or *Nadk2* KD cells versus NTC cells are at least less than 0.05 for time-points other than the “0 min” time-point. ^a^p < 0.05, *Nadk* KD vs. NTC cells; ^b^p < 0.05, *Nadk2* KD vs. NTC cells.

Figure S3. Metabolomic analysis reveals excessive AICAR generation in glucose-stimulated NADK- or NADK2-deficient cells. The NTC, *Nadk* KD and *Nadk2* KD cells were treated as described in Figure S2D, and were harvested at the indicated times for analysis of metabolites. Selected intermediates of purine metabolism are shown. The abundance of metabolite is given as the intensity of corresponding ion (*ion intensity*). Data are mean ± SD (N = 9). ^a^p < 0.05, *Nadk* KD vs. NTC cells; ^b^p < 0.05, *Nadk2* KD vs. NTC cells. (KEGG pathway identifier for the purine metabolism: hsa00230)

Figure S4. (A) The expression levels of *Prkaa1* (*white bar*) and *Prkaa2* (*black bar*) genes in the cells derived as described in Figure 2E are shown. Data are mean ± SD (N = 12). ****p < 0.001, vs. *Prkaa1* expression level in the NTC- and NTC2-expressing cells; ^††††^p < 0.001, vs. *Prkaa2* expression level in these cells. (B) The expression levels of *Prkaa1* (*white bar*) and *Prkaa2* (*black bar*) genes in the islets transfected as described in Figure 2G are shown. Data are mean ± SD (N = 12). ****p < 0.001, vs. *Prkaa1* expression level in the NTC3-transfected young mouse islets; ^††††^p < 0.001, vs. *Prkaa2* expression level in these islets.

Figure S5. Activation of AMPK in NADK- or NADK2-deficient cells and aged mouse islets after high-glucose treatment. (A) The NTC, *Nadk* KD and *Nadk2* KD cells were treated without (-) or with (+) 100 nM BAY-3827, stimulated with 16.5 mM glucose for the indicated times, and harvested for immunoblotting with antibodies to phosphorylated AMPKα (*pAMPKα*) and total AMPK (*AMPKα*). A representative experiment out of three is shown. It should be noted that the sub-figures labeled “-” are used in Figure 2D. (B) The cells were treated as described in the experimental scheme (*top panel*), and the insulin levels of culture supernatants are shown. Data are mean ± SD (N = 12). ****p < 0.001, 16.5 mM vs. 2 mM glucose treatment; ^####^p < 0.001, BAY-3827-treated vs. untreated cells receiving the 16.5 mM glucose treatment. (C) The islets isolated from the aged and young mice were treated without (*Con*) or with (*BAY-3827*) 100 nM BAY-3827, and stimulated with 2 or 16.5 mM glucose as described in the experimental scheme (*top panel*). The insulin levels of culture supernatants are shown. Data are mean ± SD (N = 12). ***p < 0.005, ****p < 0.001, 16.5 mM vs. 2 mM glucose treatment; ^††^p < 0.01, ^††††^p < 0.001, vs. young mouse islets treated with 2 mM glucose but without BAY-3827; ^§§§§^p <0.001, BAY-3827-treated vs. untreated aged mouse islets receiving the 16.5 mM glucose treatment. (D) The NTC, *Nadk* KD and *Nadk2* KD cells were treated without (-) or with (+) 5 μM compound C (*Cpd C*), stimulated with 16.5 mM glucose for various times, and harvested for immunoblotting with antibodies to phosphorylated AMPKα (*pAMPKα*) and total AMPKα (*AMPKα*). A representative experiment out of three is shown. (E) The cells were treated as described in the experimental scheme (*top panel*), and the insulin levels of culture supernatants are shown. Data are mean ± SD (N = 6). ****p < 0.001, 16.5 mM vs. 2 mM glucose treatment; ^####^p < 0.001, Cpd C-treated vs. untreated cells receiving the 16.5 mM glucose treatment. (F) The islets isolated from the aged and young mice were treated without (*Con*) or with (*Cpd C*) 5 μM Cpd C, and stimulated with 2 or 16.5 mM glucose as described in the experimental scheme (*top panel*). The insulin levels of culture supernatants are shown. Data are mean ± SD (N = 6). *p < 0.05, ***p < 0.005, 16.5 mM vs. 2 mM glucose treatment; ^†^p < 0.05, ^††††^p < 0.001, vs. young mouse islets treated with 2 mM glucose but without Cpd C; ^§^p <0.05, Cpd C-treated vs. untreated aged mouse islets receiving the 16.5 mM glucose treatment.

Figure S6. Phosphorylation of AMPK but not its expression increases in the islets of aged mouse after oral glucose challenge. Representative images show the IHC staining of phosphorylated AMPKα (*pAMPKα*) and total AMPKα (*AMPKα*) in the islets of the aged and young mice which were fed with a bolus of glucose solution for the indicated times (original magnification: 100 ×).

**SUPPLEMENTARY FIGURES**

**Figure S1.**


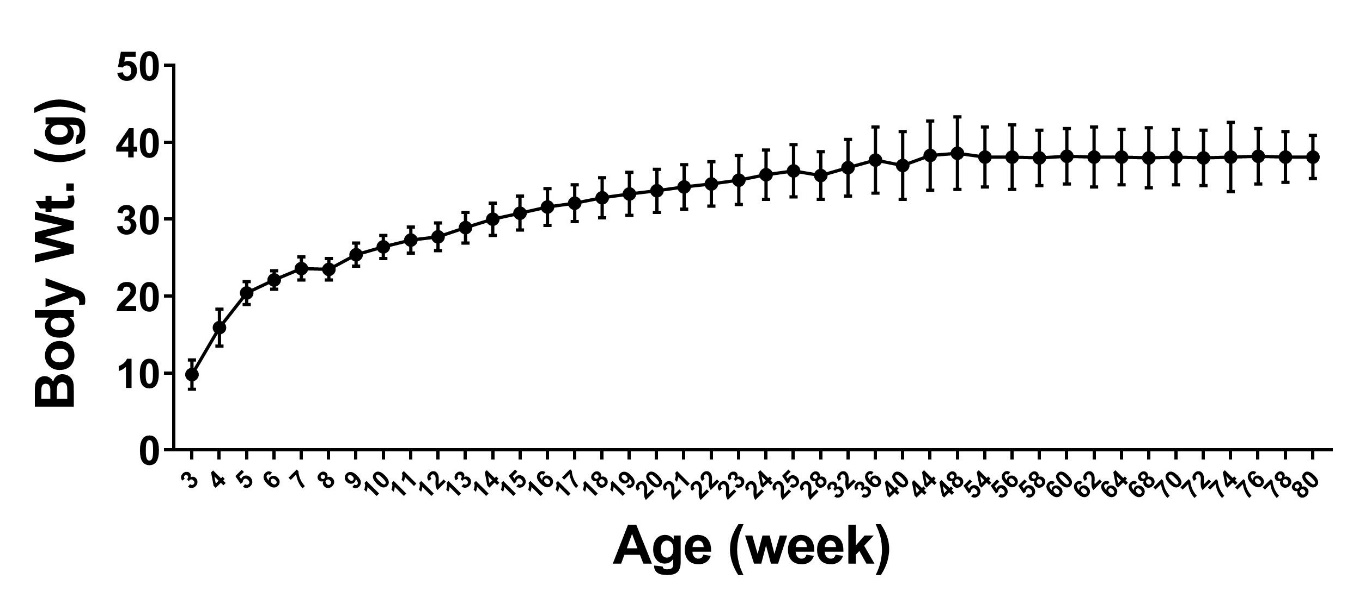


**Figure S2**

**
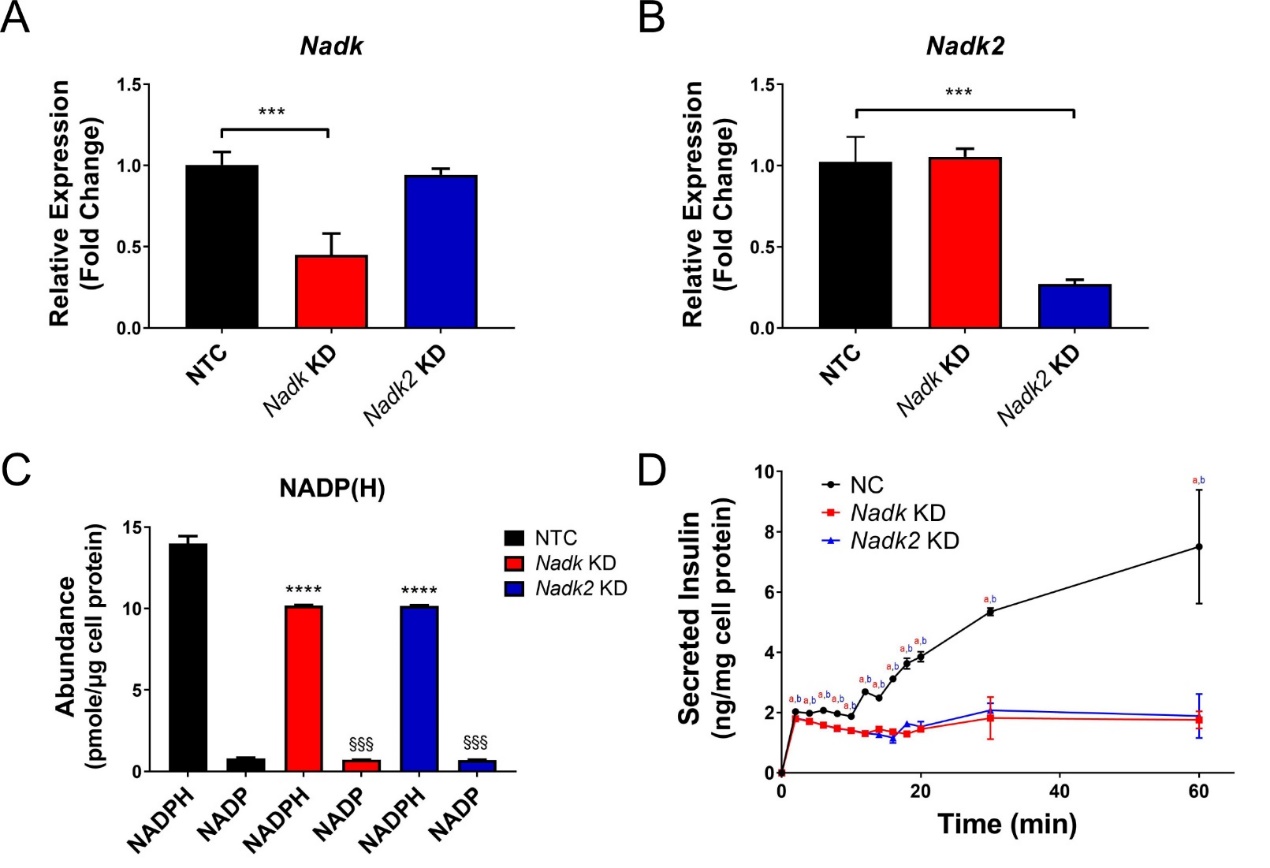
**

**Figure S3**


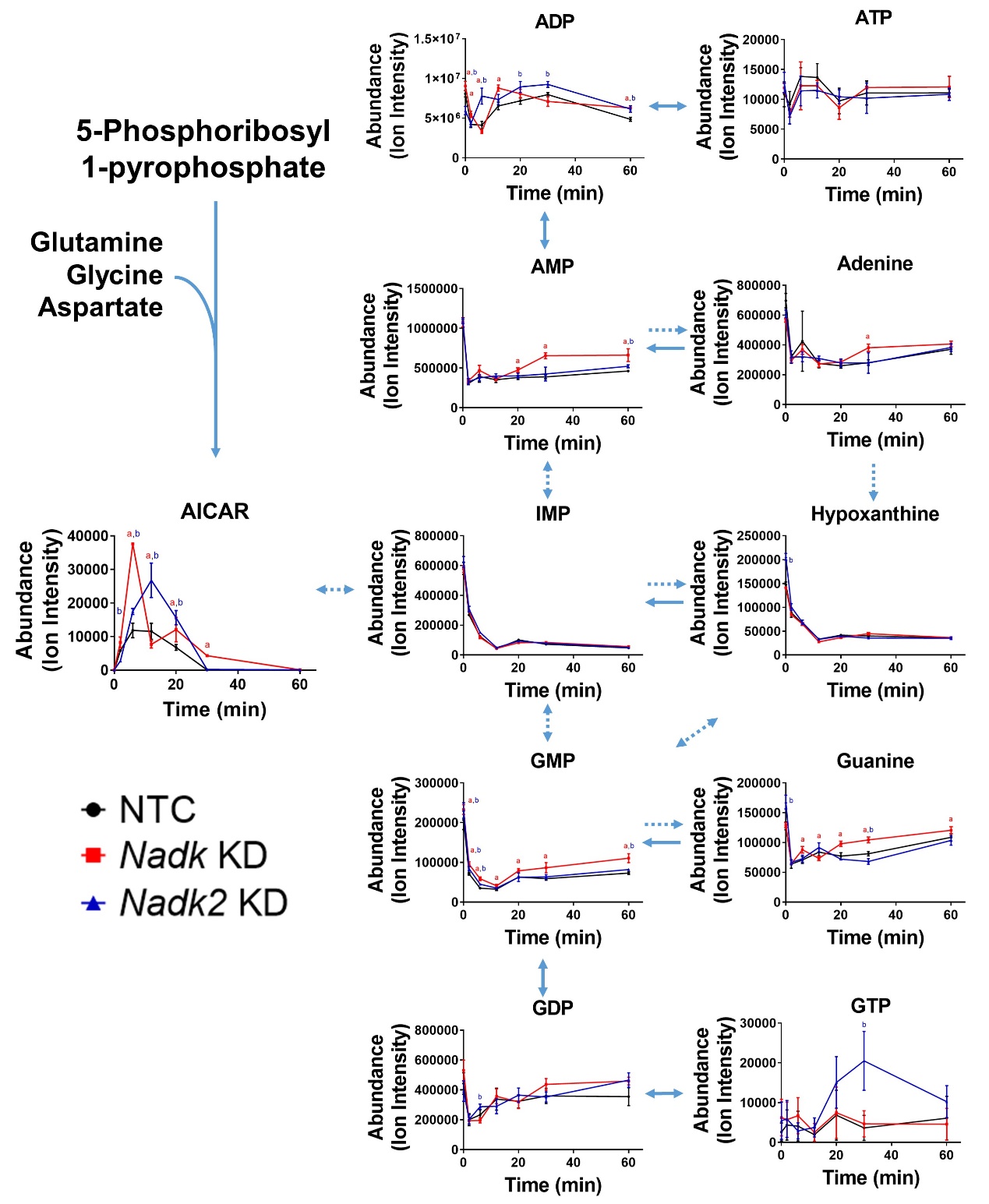


**Figure S4**


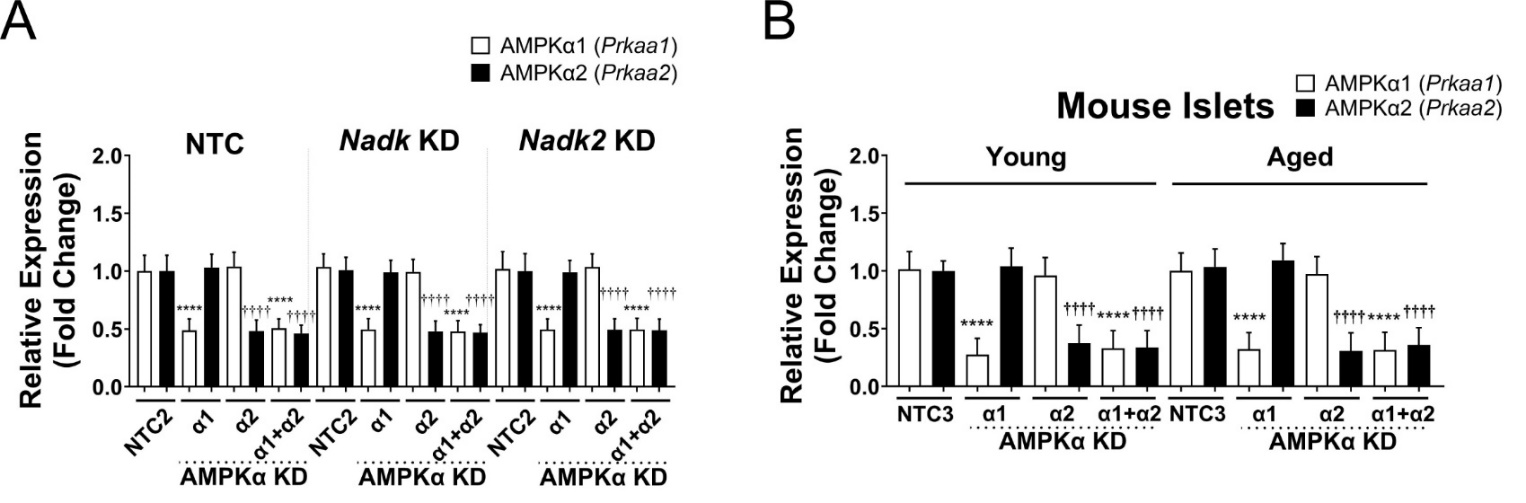


**Figure S5**


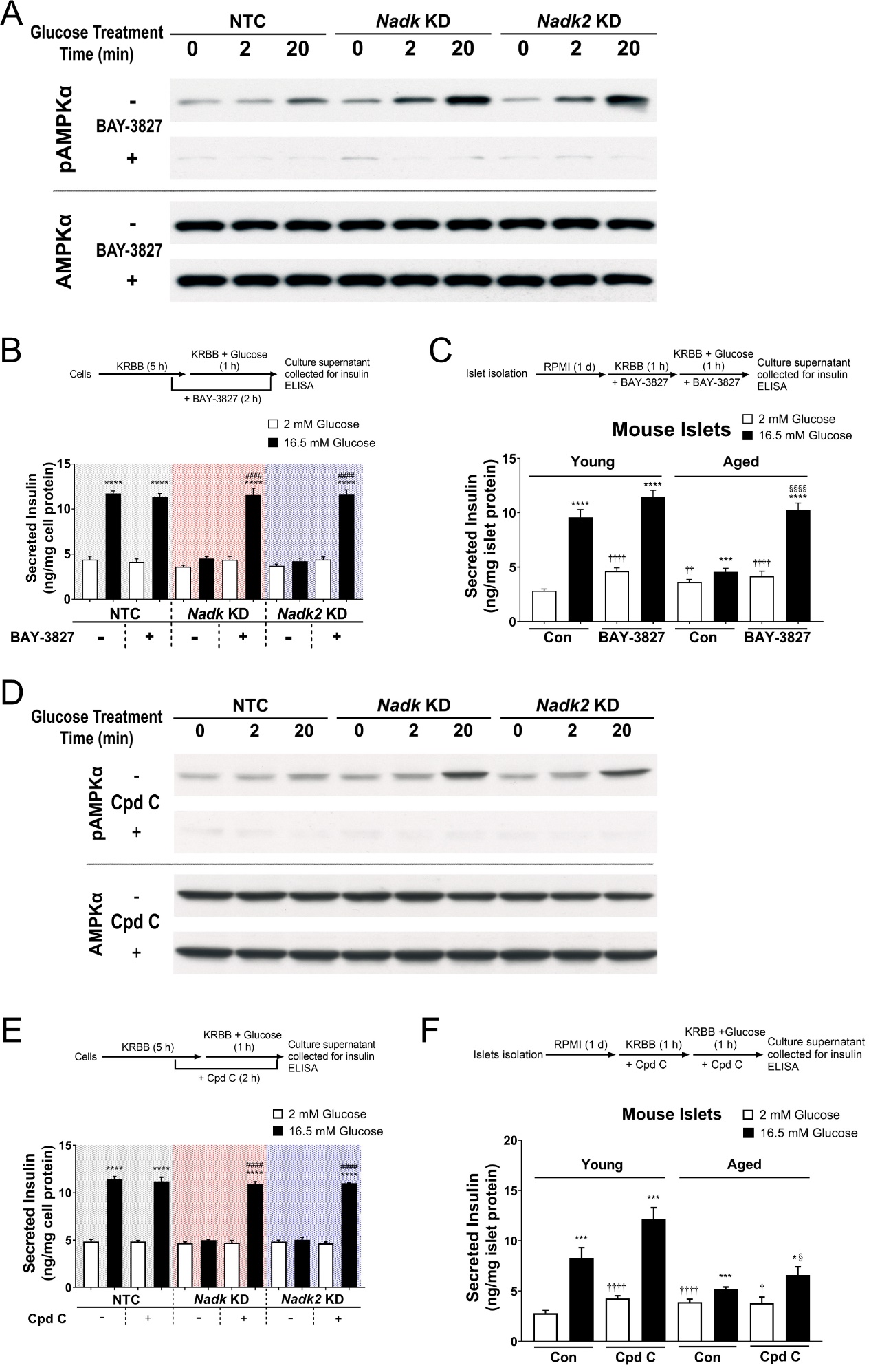


**Figure S6**

**
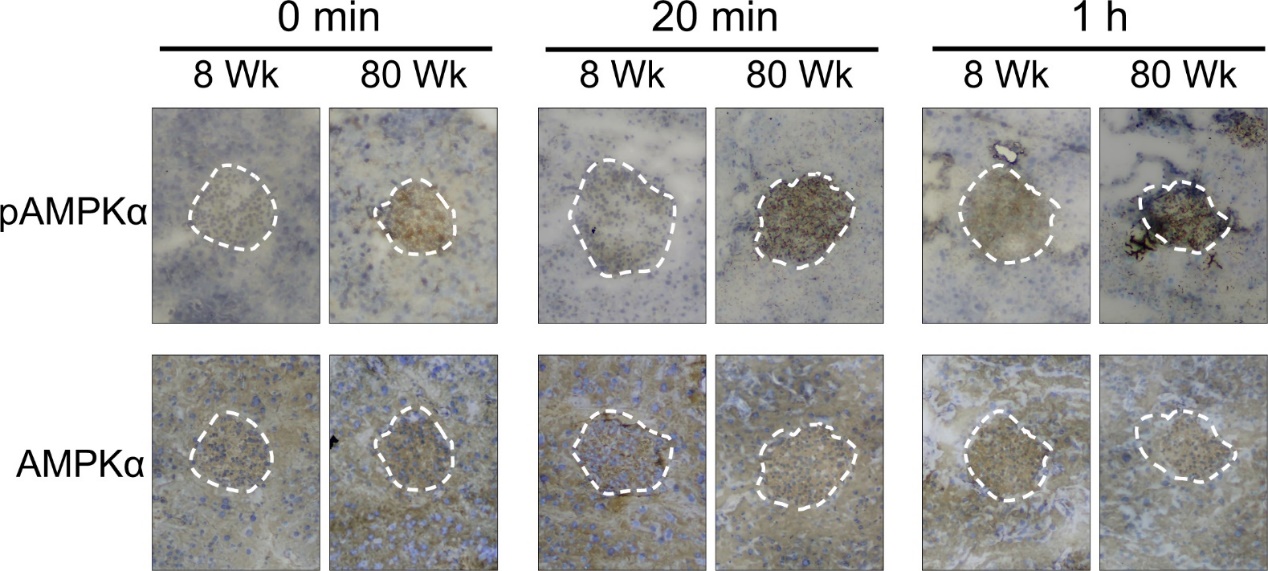
**
